# Systemic inhibition of tissue-nonspecific alkaline phosphatase alters the brain-immune axis in experimental sepsis

**DOI:** 10.1038/s41598-019-55154-2

**Published:** 2019-12-11

**Authors:** Allison L. Brichacek, Stanley A. Benkovic, Sreeparna Chakraborty, Divine C. Nwafor, Wei Wang, Sujung Jun, Duaa Dakhlallah, Werner J. Geldenhuys, Anthony B. Pinkerton, José Luis Millán, Candice M. Brown

**Affiliations:** 1 Department of Microbiology, Immunology, and Cell Biology, School of Medicine, Center for Basic and Translational Stroke Research, Rockefeller Neuroscience Institute, West Virginia University, Morgantown, WV USA; 2 Department of Neuroscience, School of Medicine, Center for Basic and Translational Stroke Research, Rockefeller Neuroscience Institute, West Virginia University, Morgantown, WV USA; 3 Department of Physiology and Pharmacology, School of Medicine, Center for Basic and Translational Stroke Research, Rockefeller Neuroscience Institute, West Virginia University, Morgantown, WV USA; 4 Department of Pharmaceutical Sciences, School of Pharmacy, Center for Basic and Translational Stroke Research, Rockefeller Neuroscience Institute, West Virginia University, Morgantown, WV USA; 5 Sanford-Burnham Prebys Medical Discovery Institute, La Jolla, CA USA

**Keywords:** Neurochemistry, Neuroimmunology, Blood-brain barrier, Neuroimmunology, Molecular medicine

## Abstract

Tissue-nonspecific alkaline phosphatase (TNAP) is a ubiquitous enzyme present in many cells and tissues, including the central nervous system. Yet its functions at the brain-immune axis remain unclear. The goal of this study was to use a novel small molecular inhibitor of TNAP, SBI-425, to interrogate the function of TNAP in neuroimmune disorders. Following intraperitoneal (IP) administration of SBI-425, mass spectrometry analysis revealed that the SBI-425 does not cross the blood-brain barrier (BBB) in healthy mice. To elucidate the role of TNAP at the brain-immune axis, mice were subjected to experimental sepsis and received either vehicle or SBI-425 (25 mg/kg, IP) daily for 7 days. While SBI-425 administration did not affect clinical severity outcomes, we found that SBI-425 administration suppressed CD4 + Foxp3+ CD25− and CD8 + Foxp3+ CD25− splenocyte T-cell populations compared to controls. Further evaluation of SBI-425’s effects in the brain revealed that TNAP activity was suppressed in the brain parenchyma of SBI-425-treated mice compared to controls. When primary brain endothelial cells were treated with a proinflammatory stimulus the addition of SBI-425 treatment potentiated the loss of barrier function in BBB endothelial cells. To further demonstrate a protective role for TNAP at endothelial barriers within this axis, transgenic mice with a conditional overexpression of TNAP were subjected to experimental sepsis and found to have increased survival and decreased clinical severity scores compared to controls. Taken together, these results demonstrate a novel role for TNAP activity in shaping the dynamic interactions within the brain-immune axis.

## Introduction

Inflammation is a vital part of the immune response and a primary component in host defense and in the resolution of disease; it exhibits both positive and negative consequences in the brain or in the periphery depending on the timing, location, and underlying mechanisms of infection, injury, or disease. The mechanisms underlying brain-immune communication are unique due, in part, to the blood-brain barrier (BBB). The BBB is a regulatory interface between the brain and systemic circulation consisting of a network of cerebral endothelial cells linked together by junctional adhesion molecules and surrounded by astrocytes, pericytes, and other supportive cells. A compromised BBB due to injury or disease commonly initiates a series of inflammatory signaling pathways which collectively result in neuroinflammation. An important component of the neuroinflammatory response is the activation of astrocytes and microglia. While a transient level of neuroinflammation is necessary for an appropriate immune response, sustained and chronic levels of inflammation lead to neurological dysfunction and disease.

There are numerous proteins that work together to orchestrate an appropriate inflammatory response in peripheral tissues and throughout the brain. Several lines of evidence suggest that the alkaline phosphatase (AP) family of enzymes may play an important role in the regulation of immunity. In an alkaline environment, AP catalyzes the hydrolysis of phosphate groups from a number of substrates^1,2^. Three of the four human AP isoenzymes are tissue specific, while the fourth and most abundant member of the family is tissue-nonspecific (i.e. TNAP). TNAP, also known as liver/bone/kidney AP, is ubiquitously expressed in numerous cell types^3^. Genetic ablation or over-expression of TNAP in humans (gene: *ALPL*) or mice (gene: *Alpl* or *Akp2*) demonstrates a defined role in bone and teeth mineralization as well as vascular calcification^4–7^. Additionally, TNAP assists with lipopolysaccharide (LPS) and nucleotide detoxification, and emerging evidence suggests an immunoregulatory role for TNAP throughout the immune system^8,9^. TNAP activity is abundant within endothelial cells and neurons of the central nervous system (CNS), and has historically been used as a marker of cerebral microvessels^10–16^. TNAP is the only isozyme found in the brain, and changes in TNAP activity in injured or diseased brains suggest a putative role in the regulation of neuroinflammation^17–20^.

Although APs were discovered nearly a century ago, surprisingly little is known about their function and mechanisms due to a lack of genetic and pharmacologic tools^21^. *Alpl* null mice only survive for approximately 10 days due to problems associated with hypophosphatasia and epileptic seizures, thus limiting studies of TNAP function to the postnatal period^22^. *Alpl*-floxed mice were recently used to confirm the importance of TNAP in mineralization of teeth and bone^23^. Previously, most pharmacological inhibitors used to interrogate TNAP function were either non-specific for TNAP isozymes or limited to *in vitro* applications, thus highlighting the need for specific inhibitors of TNAP with both *in vivo* and *in vitro* activity. 5-((5-chloro-2-methoxyphenyl)sulfonamide) nicotinamide, or SBI-425, is a novel, highly specific TNAP inhibitor^4,24^. *In vivo* studies demonstrate that SBI-425 suppresses aortic calcification in mice that overexpress TNAP in smooth muscle cells, which results in reduced aortic calcification and increased life-span^4,24^. Although the role of TNAP in the cardiac vasculature is well-described, a defined role for TNAP in the central nervous system and the immune system remains unclear.

The goal of this study was to elucidate unknown functions of TNAP at the brain-immune interface via pharmacological inhibition of the enzyme. We therefore sought to characterize the effect of SBI-425 on inhibition of murine brain TNAP enzyme activity through pharmacological, biochemical, histological, and behavioral approaches. In the first set of studies we optimized a bioassay to measure brain AP activity using *ex vivo* and *in vivo* methods of SBI-425 administration. In the second set of studies, we investigated the *in vivo* activity of SBI-425 during acute systemic inflammation by using a cecal ligation and puncture model of experimental sepsis. We hypothesized that SBI-425 administration to septic mice would suppress brain TNAP activity, enhance neuroinflammation, and promote peripheral immunosuppression in the later stages of sepsis. The results obtained from *in vivo* and *in vitro* pharmacological inhibition of TNAP enzymatic activity with SBI-425 demonstrate that the loss of TNAP’s activity during systemic proinflammatory states, i.e. sepsis, enhances disruption of the brain-immune axis. In turn, the conditional overexpression of TNAP in brain endothelial cells improves sepsis outcomes.

## Results

### *In vivo* SBI-425 administration does not cross the blood-brain barrier (BBB) in healthy mice

Since TNAP is highly expressed in cerebral microvessels, we sought to determine whether SBI-425 was capable of passing through the BBB. As a preliminary analysis, we used mass spectrometry to quantify the amount of SBI-425 detected two and eight hours following a 10 mg/kg IP injection into healthy male C57BL/6 mice. This analysis revealed low SBI-425 concentrations in plasma and homogenized brain tissue. At 2 hr post-injection the plasma level of SBI-425 was 21.6 μM and the brain level was 0.17 μM (brain:plasma <0.01); and at 8 hr post-injection the plasma level of SBI-425 was 1.26 μM and the brain level was <0.014 μM (brain:plasma <0.01) (Table 1). Low brain:plasma ratios at 2 hr and 8 hr post SBI-425 injection strongly suggests that SBI-425 does not cross the BBB under normal physiological conditions.

**Table 1:** SBI-425 concentrations in plasma and brain.

2 h post SBI-425				
	Mean	SD	CV	n
Plasma (ng/mL)	7356.667	2281.235	31.0	3
Brain (ng/g)	57.200	21.884	38.3	3
B/P ratio	0.0077	0.0009	11.5	3
**8 h post SBI-425**				
Plasma (ng/mL)	432.000	148.162	34.3	3
Brain (ng/g)	1.783	3.089	173.2	3
B/P ratio	0.0031	0.0054	173.2	3

Healthy C57BL/6J mice received IP injections of SBI-425 followed by harvest of plasma and brain at 2 hours (n = 3) and 8 hours (n = 3). SBI-425 levels in plasma and brain homogenates were quantified by liquid chromatography tandem mass spectrometry (LC/MS/MS). SD = standard deviation and CV = coefficient of variation.

B/P = brain/plasma ratio.

**Figure 1:**
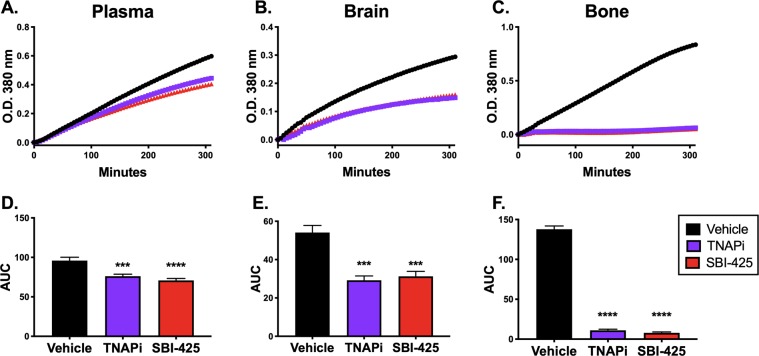
SBI-425 inhibition of TNAP activity in mouse plasma, brain, and bone homogenates. Kinetic absorbance (O.D. 380 nm) measurements were recorded over 5 hours to quantify AP enzyme activity. Plasma, brain, and bone samples were spiked with 40 µM SBI-425, 100 µM TNAP inhibitor (TNAPi), or vehicle. Curves illustrating mean absorbance from **(A)** plasma, (**B)** brain, and **(C)** bone samples are shown. The area under the curve (AUC) was calculated to determine the level of TNAP activity in each sample. Spike-in of TNAPi or SBI-425 in all samples equally inhibited TNAP activity in **(D)** plasma (P < 0.0001), **(E)** brain (P = 0.0001), and **(F)** bone (P < 0.0001). Data represent mean ± SEM and were analyzed with one-way ANOVA followed by Dunnett’s multiple comparisons tests showing treatment results compared to vehicle; n = 4–12 samples from individual mice per group. Asterisks represent multiple comparisons: *P ≤ 0.05; **P ≤ 0.01; ***P ≤ 0.001; and ****P ≤ 0.0001.

The next set of experiments addressed whether SBI-425 could inhibit brain AP activity *in vitro*. We collected plasma, brain, and bone tissue from healthy male C57BL/6 mice following saline perfusion. Plasma and supernatants from tissue homogenates were spiked with 40 μM SBI-425, 100 μM of an early TNAP inhibitor (TNAPi), or vehicle (DMSO) and AP activity was kinetically read for 5 hours. TNAPi has been previously described; the *in vitro* efficacy is similar to SBI-425 but due to its biochemical properties it cannot be used *in vivo*^24,25^. There was a significant decrease in AP activity in all TNAPi- and SBI-425-treated samples compared to vehicle (Fig. 1). This decrease in TNAP activity was highest in bone (Fig. 1C,F) compared to significant reductions in brain homogenate and plasma (Fig. 1A,B,D,E), thus indicating that SBI-425 is a potent TNAP inhibitor *in vitro*.

### SBI-425 displays *in vivo* TNAP inhibitory activity in plasma and brain

**Figure 2:**
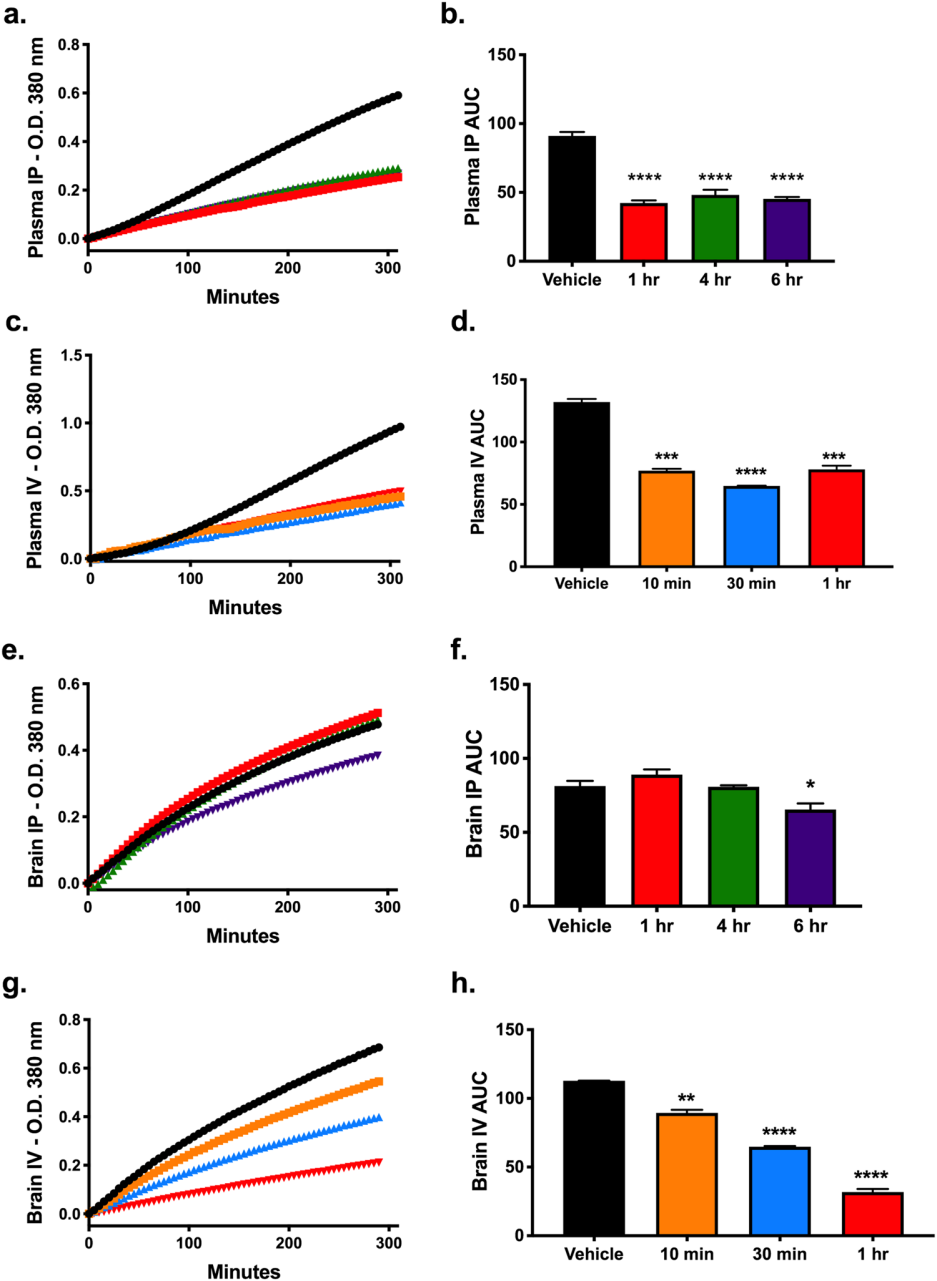
SBI-425 kinetics in plasma and brain via intraperitoneal and intravenous administration. Male and female C57BL/6 J were randomly assigned to intraperitoneal (IP; 25 mg/kg; n = 3–4/group) or intravenous (IV; 5 mg/kg; n = 2–3/group) injection groups prior to administration of vehicle or SBI-425. IP-injected mice were harvested at 1, 4, or 6 hours following treatment and IV-injected mice were harvested 10, 30, or 60 minutes following treatment. Kinetic absorbance measurements were recorded at O.D. 380 nm and curves (mean absorbance) are shown for each dosing paradigm: **(a)** plasma IP, **(b)** plasma IV, **(c)** brain IP, and **(d)** brain IV. TNAP activity was calculated from the area under the curve (AUC, mean ± SEM) for **(e)** plasma IP (P < 0.0001), **(f)** plasma IV (P < 0.0001), **(g)** brain IP (P = 0.0019), and **(h)** brain IV (P < 0.0001). Results were analyzed with one-way ANOVA followed by Dunnett’s test with all comparisons made to vehicle treatment. Asterisks represent multiple comparisons: *P ≤ 0.05; **P ≤ 0.01; ***P ≤ 0.001; and ****P ≤ 0.0001.

Given that our results showed that SBI-425 was able to inhibit brain TNAP activity *in vitro*, but unable to cross the BBB *in vivo*, we then tested whether SBI-425 treatment could inhibit TNAP activity *in vivo* via different routes. We administered a single dose of SBI-425 or vehicle solution (10% DMSO, 10% Tween-80, 80% water) to healthy C57BL/6J mice by either intraperitoneal (IP) or retro-orbital (IV) injection. One group of mice were injected IP with a 25 mg/kg dose of SBI-425 or vehicle, followed by plasma and brain tissue harvest at 1, 4, or 6 hours post-injection. A second group of mice were injected IV with a 5 mg/kg dose of SBI-425, followed by plasma and brain harvest at 10, 30, or 60 mins post-injection. Timepoints for tissue collection were different between the two groups since we reasoned that IV injected SBI-425 would require less time to reach the brain than IP administered SBI-425. Our results show that TNAP activity is inhibited by SBI-425 in plasma at all time-points for both IP (Fig. 2a,b) and IV injections (Fig. 2c,d). However, IP-injection of SBI-425 inhibited TNAP activity in brain homogenate at 6 h post-injection (Fig. 2e,f), while IV-injection of SBI-425 exhibited a time-dependent inhibition of TNAP activity (Fig. 2g,h).

### TNAP inhibition does not alter survival or behavioral outcomes in experimental sepsis

**Figure 3:**
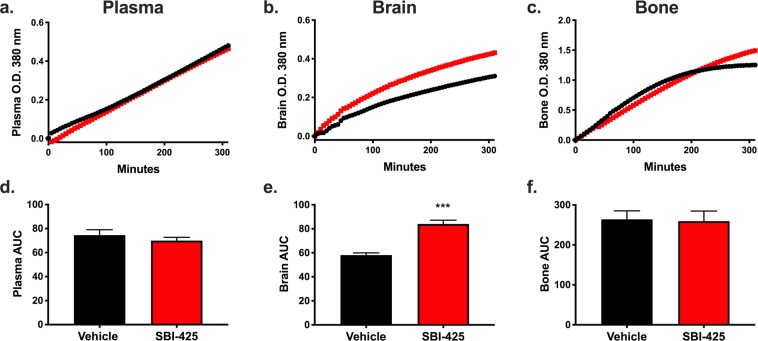
SBI-425-mediated inhibition of plasma, brain, and bone TNAP activity in murine experimental sepsis. Kinetic absorbance (O.D. 380 nm) measurements of TNAP activity are depicted in curves (showing mean absorbance) for **(a)** plasma, **(b**) brain, and (**c**) bone harvested from mice that were subjected to cecal ligation and puncture (CLP) and treated with either vehicle (n = 5) or SBI-425 (n = 3) every 24 hours for 7 days. TNAP activity levels between vehicle and SBI-425 groups were compared measuring area under the curve (AUC), reported as means ± SEM, and analyzed by Student’s t-test in **(d)** plasma, **(e)** brain (p = 0.0002), and **(f)** bone; ***P ≤ 0.001.

To determine SBI-425’s effects on the brain-immune axis *in vivo*, female C57BL/6 mice were subjected to cecal ligation and puncture (CLP), a mouse model of experimental sepsis. Each mouse received SBI-425 (25 mg/kg, IP) or vehicle injections one hour post-CLP surgery. SBI-425 or vehicle injections continued once daily for a total of 7 days, followed by plasma and tissue collection 24 hr after the final vehicle or SBI-425 injection. No differences were observed in plasma or bone AP activity levels, but there was a significant increase in brain AP activity in SBI-425-treated mice (Fig. 3).

**Figure 4:**
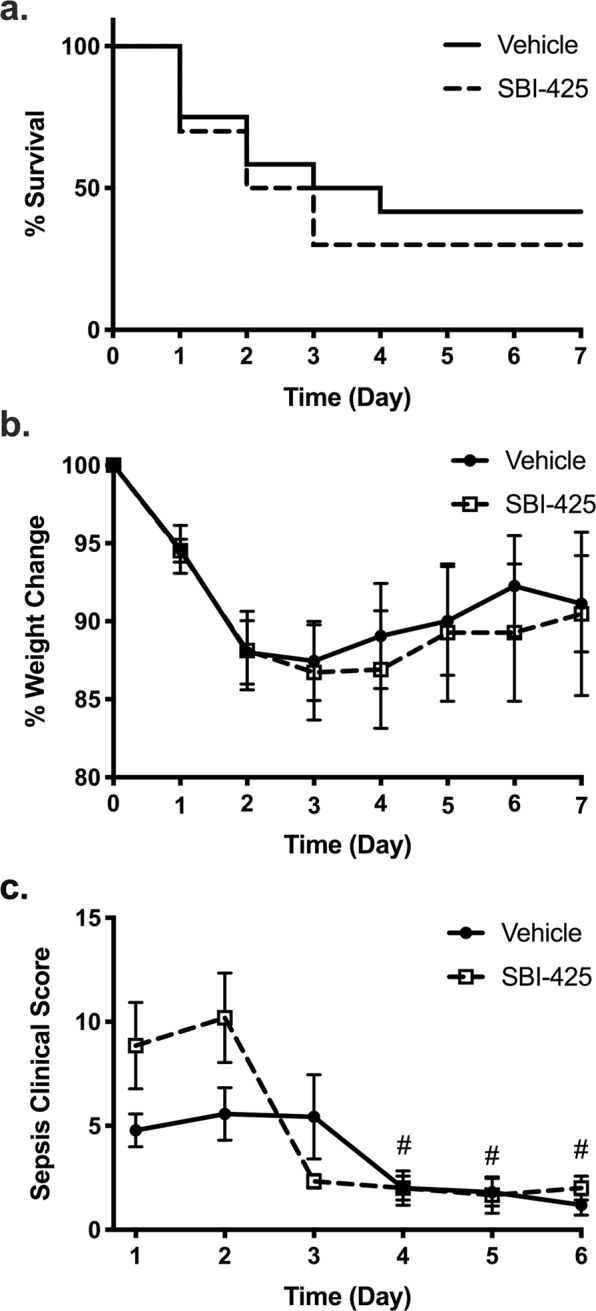
Pharmacological inhibition of TNAP does not alter survival, weight loss, or clinical severity scores in experimental sepsis. Mice were subjected to cecal ligation and puncture (CLP) and were randomly assigned to treatment with either vehicle (n = 12) or SBI-425 group (n = 10) on day 0. **(a)** Survival, **(b)** weight change, and **(c)** clinical scores were assessed daily following CLP surgery. Mice were treated once daily with 25 mg/kg (IP) SBI-425 or equivalent volume of vehicle for 7 days. Survival analysis was performed by the log rank Mantel-Cox test. Weight change and clinical severity scores were analyzed by mixed effects model followed by Dunnett’s test. Clinical scores showed an overall interaction between time and SBI-425 treatment (P = 0.0035) and a main effect of time (P = 0.001), while post hoc analyses showed that clinical scores at days 4,5 and 6 were significantly different compared to day 1 for SBI-425-treated mice; # represents P ≤ 0.05 compared to the SBI-425 score at day 1. Data represent means ± SEM.

Next, due to TNAP’s role in LPS and nucleotide detoxification, we hypothesized that SBI-425 administration would increase mortality and worsen clinical severity. Daily observations of all mice showed that there were no significant differences in mortality (Fig. 4a) or weight loss (Fig. 4b) in vehicle and SBI-425 treated mice over 7 days. Sepsis clinical severity scores, obtained using a murine sepsis severity scoring paradigm, decreased over time (Fig. 4c). Likewise, no differences in locomotor activity were observed between vehicle or SBI-425 treated mice (Table 2). These results demonstrate that SBI-425 administration did not potentiate any effects of CLP injury for these clinical outcomes.

**Table 2:** Open field behavior in CLP-injured mice treated with vehicle or SBI-425.

	Vehicle Beam Breaks (mean ± SEM)	SBI-425 Beam Breaks (mean ± SEM)	P value
Total horizontal movement**s**	1968 ± 471	1933 ± 407	0.96
Total peripheral movement**s**	1519 ± 360	1335 ± 351	0.79
Total central movement**s**	449 ± 121	598 ± 88	0.40
Total rearing movements	72 ± 24	54 ± 30	0.64

Surviving CLP-injured mice treated with vehicle (n = 6) or SBI-425 (n = 4) underwent open field behavior testing on day 2 post-surgery. Movements were recorded in each plane based on the number of infrared beam breaks. No significant differences were seen in total horizontal, peripheral, central, or rearing movements between vehicle and SBI-425 treated mice.

### TNAP inhibition during experimental sepsis suppresses splenic Foxp3^+^ T-cells

**Figure 5:**
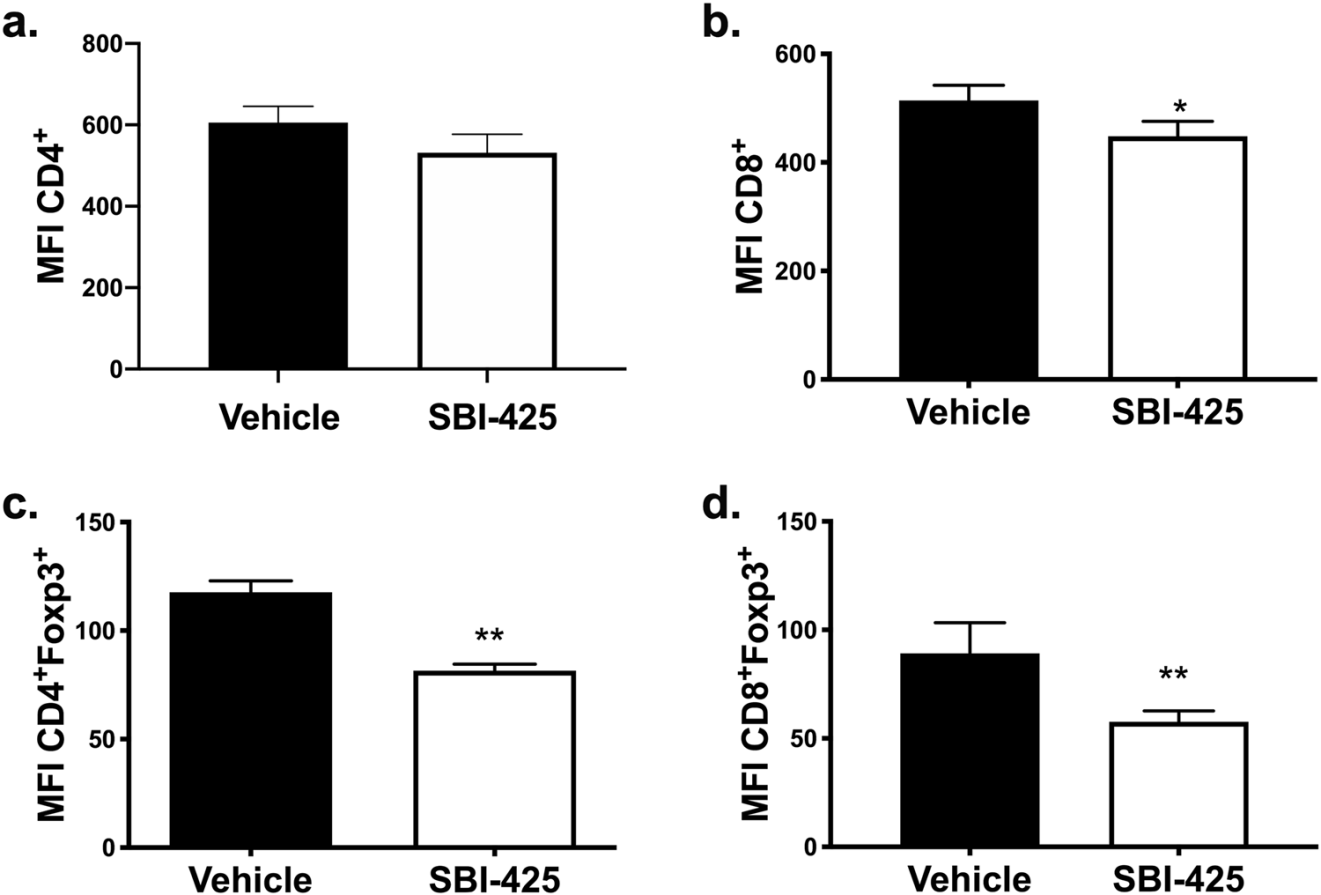
SBI-425 treatment suppressed Foxp3 expression in CD4^+^ and CD8^+^ T cells in splenocytes from CLP-injured mice. Spleens were harvested on day 7 post-CLP, followed by isolation and purification of splenocytes. Splenocyte populations in vehicle (n = 5) and SBI-425 (n = 3) treated mice were immunophenotyped by flow cytometry and reported as median fluorescent intensity (MFI). **(a)** CD4^+^ T cell MFI did not differ between vehicle and SBI-425 splenocytes (P = 0.28), while **(b)** CD8^+^ T cell MFI was lower in splenocytes from SBI-425- than vehicle-treated mice (P = 0.017). Foxp3+ MFI was also lower in **(c)** CD4^+^Foxp3^+^CD25^−^ (P = 0.0027) and **(d)** CD8 + Foxp3+ CD25^−^ (P = 0.010) T cell populations from SBI-425-treated mice compared to vehicle-treated mice. Data were analyzed by Student’s t-test and represent means ± SEM; **P ≤ 0.01.

To determine whether inhibition of TNAP affects the immune response in late sepsis, splenocytes were isolated from each mouse and immunophenotyped using flow cytometry. Murine splenocytes were gated using the strategy shown in Supplementary Fig. 1 and the median fluorescent intensity (MFI) of staining for each marker was calculated. Vehicle- and SBI-425-treated mice had similar staining intensities for CD4^+^ T cells (Fig. 5a), while SBI-425 splenocytes had a lower staining intensity for CD8^+^ T cells than vehicle splenocytes (Fig. 5b). To determine whether SBI-425 altered regulatory T cell populations (Tregs) in late sepsis, CD4^+^ and CD8^+^ T cells were gated for CD25 and Foxp3, which are two prototypical markers for Tregs^26,27^. All CD4^+^ and CD8^+^ T cells isolated from splenocytes were CD25^−^ and Foxp3^+^. SBI-425 decreased the MFI of Foxp3+ T cell subsets. Both CD4^+^Foxp3^+^ (Fig. 5c) and CD8^+^Foxp3^+^ (Fig. 5d) T cell subsets were significantly decreased in splenocytes from SBI-425-treated mice compared to their vehicle counterparts.

### SBI-425 suppresses alkaline phosphatase enzyme activity in brain parenchyma

**Figure 6:**
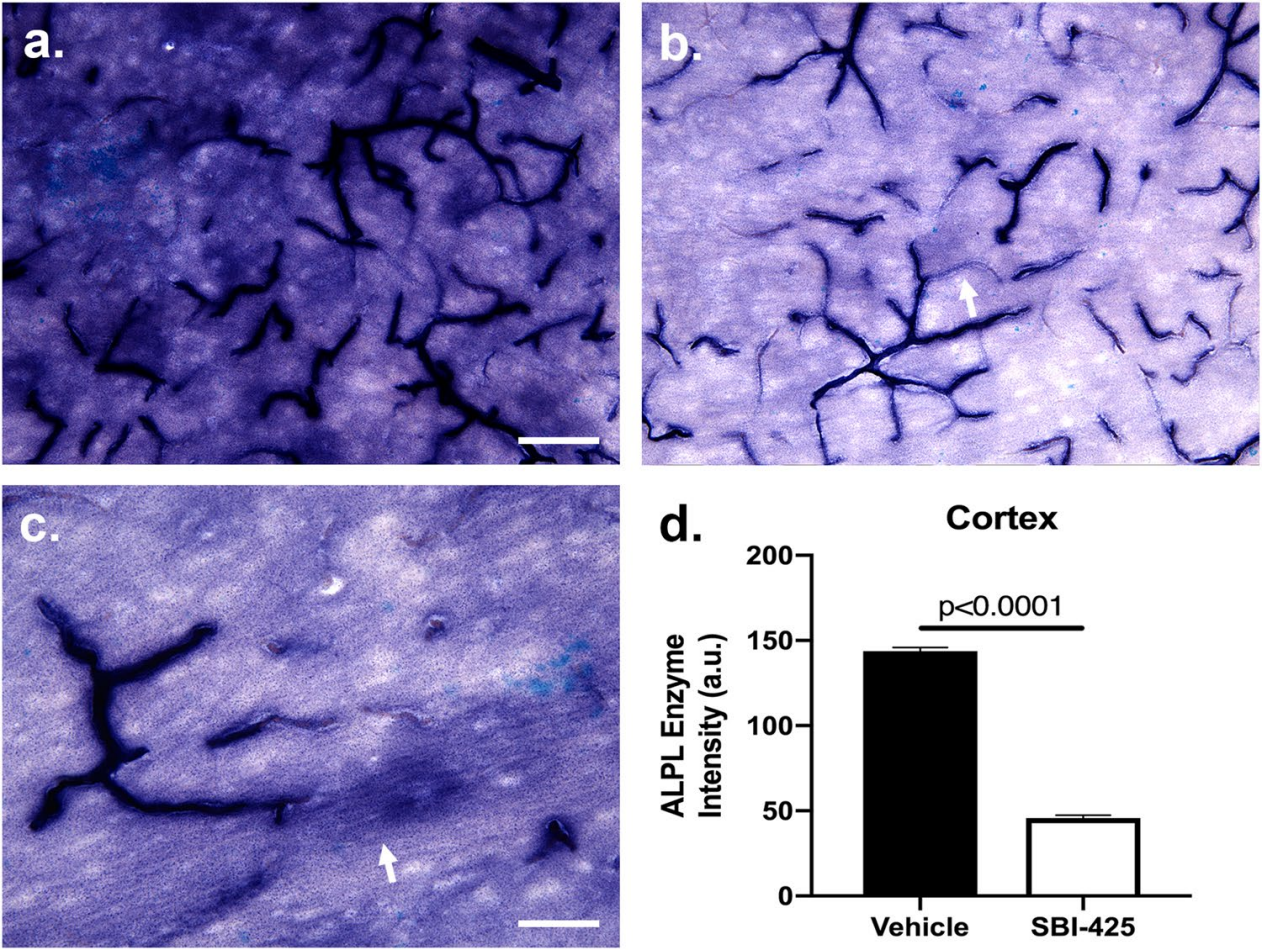
Alkaline phosphatase enzyme activity in the brains of septic mice treated with vehicle or SBI-425. Sepsis increased TNAP enzyme activity in the (**a**) parenchyma of vehicle treated mice (n = 5) that was inhibited by (**b**) SBI-425 treatment (n = 3). The maximal intensity of blood vessel staining was similar in both groups although vessels with lighter staining were observed in both treatment groups (arrow in **b**). Detailed examination of the neural parenchyma reveals enzyme activity in the processes of neurons (arrow in **c**) but not the cell body. (**d**) Graphical analysis of mean staining intensity analyzed by Student’s t-test revealed significant differences in parenchymal staining between treatment groups (P < 0.0001). Scale bar in (**a**,**b)** = 80 μm. Scale bar in (**c**) = 40 μm. Data represent means ± SEM; ****P ≤ 0.0001.

To determine whether SBI-425 decreased *in vivo* brain TNAP activity, AP enzyme activity assays were performed on brain tissue sections harvested at day 7 from the same set of vehicle and SBI-425 treated mice. Areas of detectable AP enzyme activity were denoted by the presence of a blue-purple stain. AP enzyme activity was localized to blood vessels and parenchymal neuronal fibers in the brains of septic mice treated with vehicle or SBI-425 (Fig. 6). Sepsis resulted in intense dark blue staining of the neuropil (Fig. 6a) compared to mice treated with SBI-425 (Fig. 6b). The maximal staining intensity of blood vessel was similar in both treatment groups; however, regions of lighter staining were observed in all sections of both treatments (arrow in b). High-magnification microscopy revealed the cell bodies of neurons appear relatively unstained while processes can be identified traveling through the parenchyma and white matter (arrow in Fig. 6c). Quantification of AP cortical enzyme activity revealed a significant reduction in parenchymal AP staining intensity in SBI-425-treated septic mice compared to septic mice who received vehicle (Fig. 6d).

### TNAP inhibition does not alter astrocyte or microglial activation

**Figure 7:**
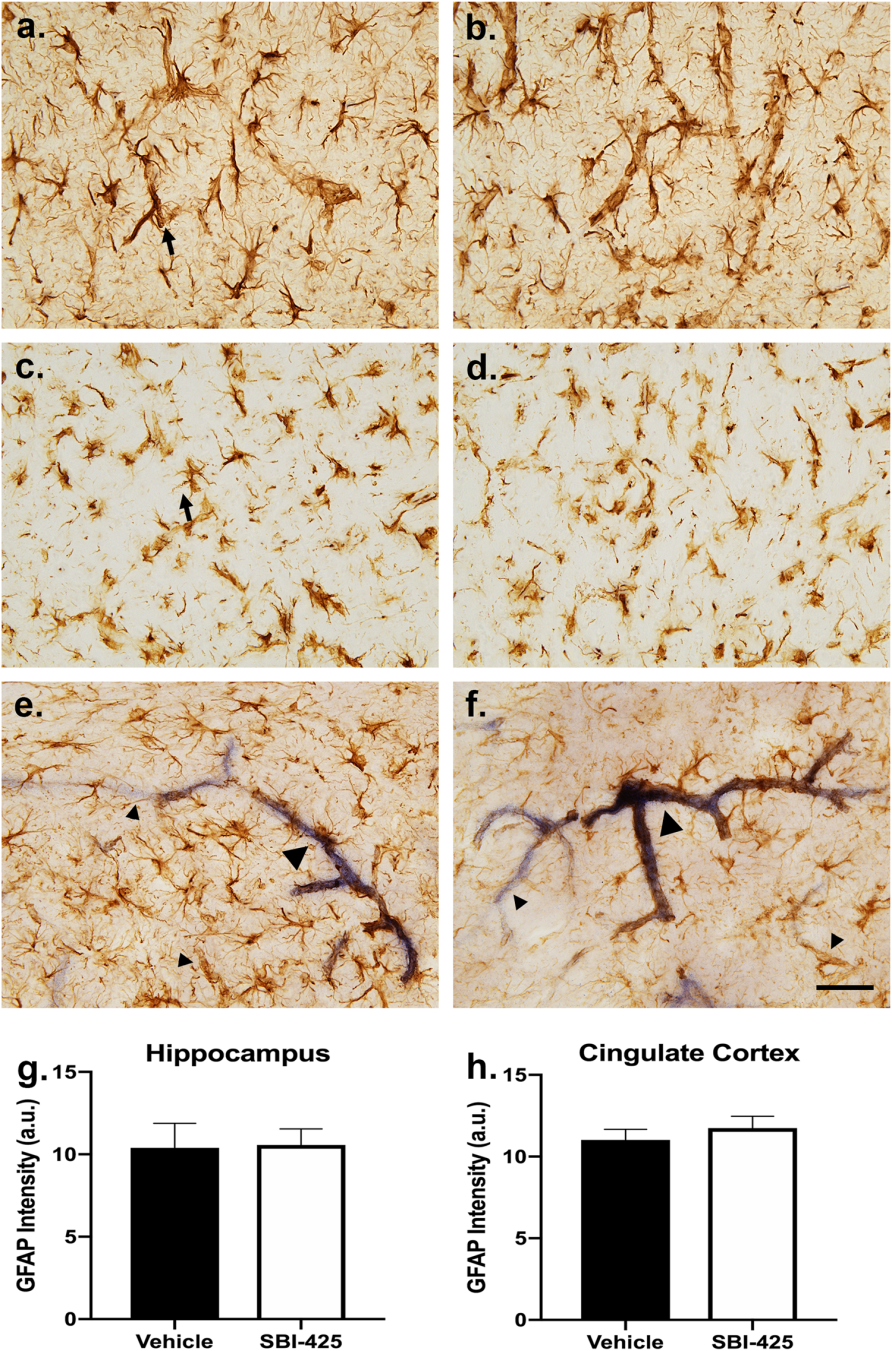
Astrocyte immunoreactivity is affected by sepsis but not SBI-425 treatment. (**a–d**) Astrocytes in both gray and white matter displayed morphology of activation following sepsis. (**a**,**b**) Protoplasmic astrocytes displayed intense immunoreactivity in the cell body and several processes while other processes remained thin and lightly stained. These astrocytes were more often seen in apposition to blood vessels. (**c**,**d**) Fibrous astrocytes were intensely stained, but cell processes were more restricted to the proximal vicinity of the cell body. Sepsis treatment also resulted in the appearance of small, clear vesicles in the processes of reactive glia (arrows in **a**,**c**). (**e**,**f**) Double-staining of sections with GFAP immunohistochemistry and TNAP enzyme activity revealed co-localization of astrocyte immunoreactivity and alkaline phosphatase enzyme activity on regions of blood vessels (large arrowheads), while other vessels displayed staining for either GFAP or TNAP enzyme activity (small arrowheads). No differences in double-staining were observed following vehicle (n = 5) or SBI-425 (n = 3) treatment. Student’s t-test revealed similar levels of immunoreactivity and no differences in GFAP staining density between vehicle and SBI-425 treatment groups in the (**g**) hippocampus or (**h**) cingulate cortex. Scale bar in (**a–f**) = 40 μm. Data represent means ± SEM.

Since SBI-425 administration suppressed TNAP activity in the brain parenchyma and TNAP has an emerging role in the immune system, the next set of histological studies addressed whether SBI-425 treatment increased indices of sepsis-associated neuroinflammation. Astrocyte (GFAP) and microglial (Iba-1) activation were quantified as indices of neuroinflammation. Astrocytes responded to sepsis with an increase in GFAP immunoreactivity in the hippocampus and primary white matter tracts (Fig. 7). The morphology of reactive astrocytes was different for protoplasmic and fibrous glia. Gray matter (protoplasmic) astrocytes displayed intense immunoreactivity in the cell body and several short, thick processes (Fig. 7a,b), while other processes were thinner and lightly stained. Reactive astrocytes were observed extending processes to blood vessels. In white matter (fibrous) astrocytes of the cingulum, astrocyte cell bodies were also intensely stained, but processes appeared thicker and more restricted to the region proximal to the cell body (Fig. 7c,d). Glial-wrapped blood vessels were seen less frequently in white matter. Both types of reactive astrocytes contained small, clear vesicles in the processes (arrow in a, c). Double-staining with immunohistochemistry and TNAP enzyme activity revealed co-localization of some, but not all, cerebral vessels (Fig. 7e,f). Many regions of vessels displayed high levels of enzyme activity and intense astrocyte immunoreactivity (large arrowheads in e, f), while other regions displayed either enzyme activity or GFAP immunoreactivity (small arrowheads in e, f). No differences in double-staining were observed following treatment with SBI-425. Densitometric analysis of GFAP immunoreactivity revealed similar intensities of immunoreactivity in hippocampus (Fig. 7g) and cingulate cortex (Fig. 7h). In spite of the changes in astrocyte morphology and TNAP activity in cerebral microvessels, SBI-425 treatment had no further effect on changes in GFAP immunoreactivity induced by sepsis.

**Figure 8:**
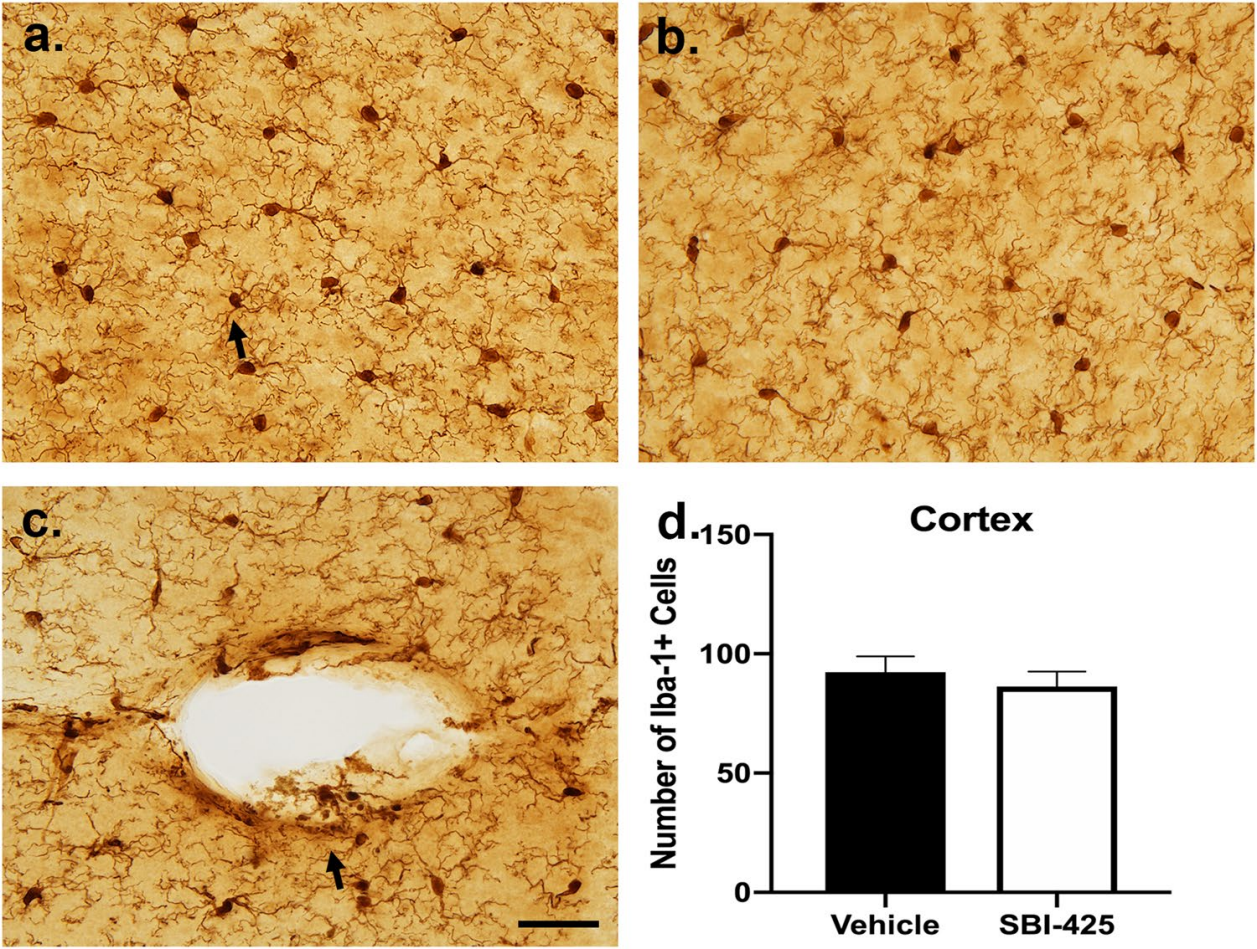
Microglial immunoreactivity is affected by sepsis but not SBI-425 treatment. The microglial response to sepsis surgery and SBI-425 treatment was largely resolved by the time-point of sacrifice. **(a)** Microglia displayed a surveillance-type morphology with a round to slightly oval cell body and many long, thin twisted processes (arrow). (**b**) Treatment with SBI-425 had no effect on microglial activation status. (**c**) Residual pathology was observed in the vicinity of blood vessels, where microglia had a round cell body with few and thicker processes that contained spheroids. Phagocytic debris was observed in microglial processes in the parenchyma proximal to and within the walls of blood vessels (arrow). (**d**) Microglial number was quantified from sections of both treatment groups and Student’s t-test revealed no significant differences in the number of glial cell bodies in the cortex between vehicle (n = 5) and SBI-425 (n = 3) treated mice. Scale bar (**a–c**) = 40 μm. Data represent means ± SEM.

Similar results were observed for microglial activation. Microglia in most brain regions of both treatment groups appeared in the surveillance state (Fig. 8). These cells had a round to oval cell body with long, thin processes that twisted through the neural parenchyma (Fig. 8a,b). In larger diameter blood vessels, however, sepsis-induced activation of microglia was persistently observed (Fig. 8c). These cells appeared round with a few short, thick processes, and contained phagocytic debris. The quantity of microglial cells was evaluated in images of medial orbital cortex in septic mice treated with vehicle or SBI-425. No differences in microglial number were observed between treatment groups (Fig. 8d).

### SBI-425 suppresses BBB integrity in brain microvascular endothelial cells

**Figure 9:**
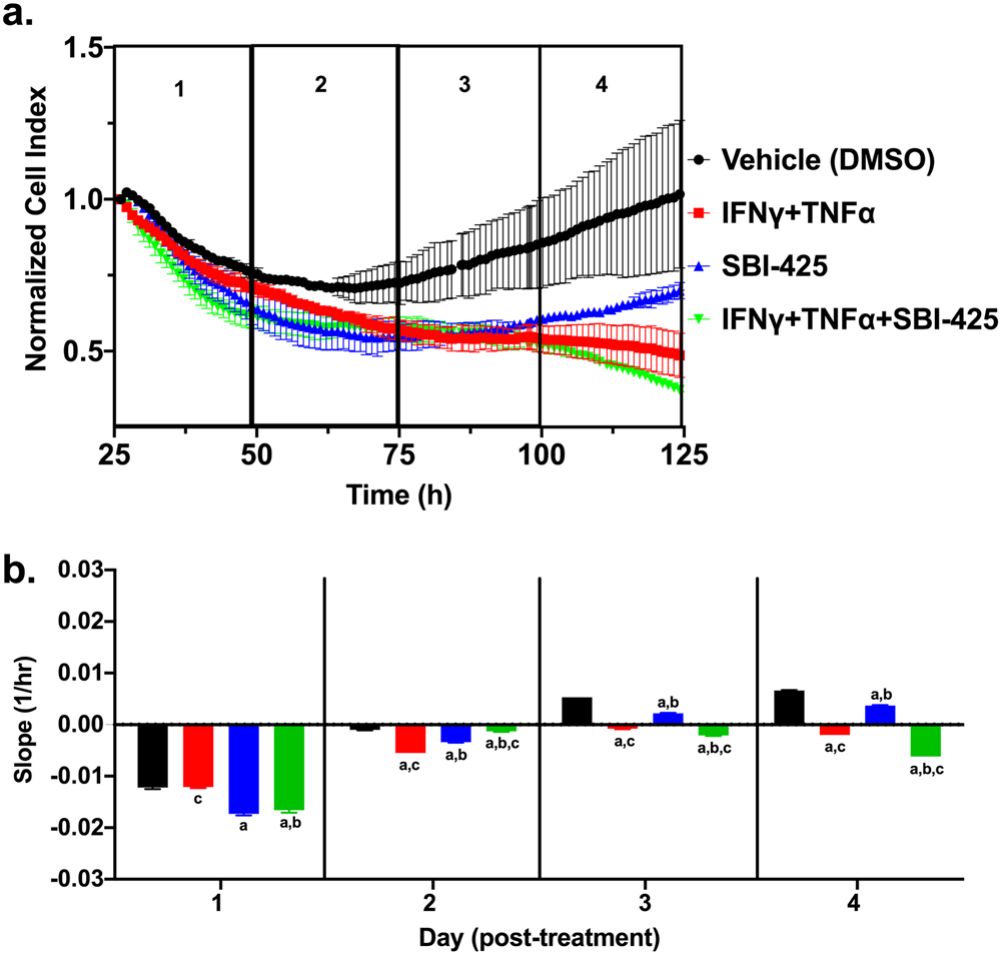
SBI-425 treatment decreases barrier function in murine brain microvascular endothelial cells (BMECs). Normalized cell index (CI) is an impedance-based measure of barrier function. (**a**) Primary murine BMECs were treated with either vehicle, IFNγ (10 ng/ml) + TNFα (10 ng/ml), SBI-425 (100 μM) or SBI-425 in combination with IFNγ + TNFα. Representative traces depicting the normalized CI (set to 1.0) at the time of treatment (25 hr after seeding cells) and recorded until the end of the experiment (125 hr after seeding cells). Each representative trace depicts means ± SD and is separated into four consecutive 24 hr intervals, designated 1–4. **(b)** Changes in the slope, which represents change in CI over each 24 hr interval, are shown over a four day period. Data represent means ± SD and were analyzed by one-way ANOVA followed by Tukey’s test with P ≤ 0.001 as significant where “*a”* indicates significance compared to vehicle; “*b”* indicates significance compared to IFNγ + TNFα; “*c”* indicates significance compared to SBI-425.

The reduction of AP activity in brain parenchyma suggested that SBI-425 is able to cross the BBB during systemic pro-inflammatory disease states (e.g. sepsis) when its integrity is compromised. Therefore, the next set of experiments addressed whether SBI-425 potentiates the effects of a proinflammatory stimulus on barrier function in murine brain microvascular endothelial cells (BMECs). To test this, real-time changes in barrier function were quantified in primary BMECs by measuring changes in cellular impedance, denoted as the cell index (CI). After reaching confluency, BMECs were treated with vehicle (DMSO) alone, SBI-425 alone, the inflammatory stimuli interferon-γ (IFNγ) combined with tumor necrosis factor-α (TNFα), or SBI-425 in combination with IFNγ and TNFα (IFNγ + TNFα). In a separate set of experiments, we confirmed that treatment with SBI-425, IFNγ + TNFα, or the combination of both treatments did not result in any significant differences in cytotoxicity (data not shown). Normalized CI was continuously recorded for ~100 hours post-treatment (Fig. 9a). Changes in the CI slope over four consecutive 24-hour intervals (i.e. four days) showed that early treatment (day 1) with SBI-425 decreased barrier function either alone or in combination with IFNγ + TNFα treatment. Smaller decreases in barrier function persisted through day 2, followed by recovery of barrier function in SBI-425 only treated cells over days 3 and 4. In contrast, the detrimental effects of IFNγ + TNFα alone or IFNγ + TNFα in combination with SBI-425 persisted until day 4 (Fig. 9b).

#### Overexpression of TNAP in brain endothelial cells improves sepsis outcomes

The final set of experiments addressed the effects of sepsis on TNAP expression and enzyme activity. Since TNAP is expressed in multiple cell types within the brain, we first tested the mRNA expression of *Alpl* in primary BMECs. Cells were treated with a vehicle (DMSO), lipopolysaccharide (LPS, 100 ng/ml), TNAP inhibitor (TNAPi, 100 μM), or a combination of LPS + TNAPi. The effects of a 100 μM dose of TNAPi on barrier function were identical to SBI-425 (data not shown.) *Alpl* expression was quantified at 3, 6, and 24 hr after treatment and was found to be elevated with LPS + TNAPi treatment at all timepoints (Supplementary Fig. 2).

**Figure 10:**
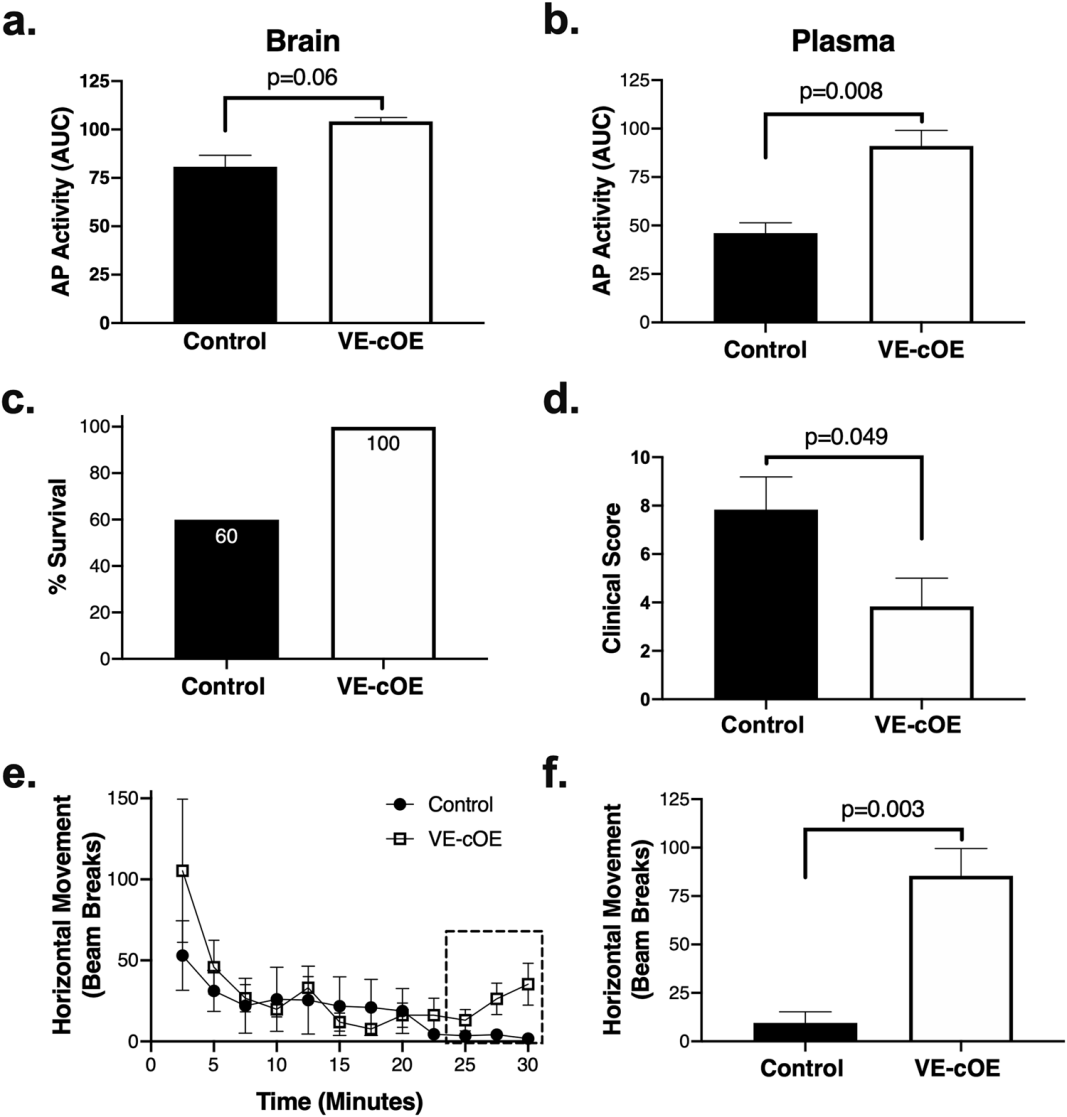
Genetic over-expression of TNAP on endothelial cells reduces morbidity and mortality after sepsis. Sham-injured VE-cOE mice have increased AP activity compared to sham-injured control animals in **(a)** brain (n = 2 per group) and **(b)** plasma (n = 3–4 per group). **(c)** Survival is increased up to 48 hr after sepsis in VE-cOE mice (survival in 6 of 6 mice) compared to controls (survival in 3 of 5 mice). **(d)** Clinical severity scores were decreased in VE-cOE mice 24 hr after sepsis (n = 6 mice per group). **(e–f)** Open field testing shows that there is increased voluntary movement by septic VE-cOE mice (n = 6) compared to controls (n = 4) in the last ten minutes of the test. Data represent means ± SEM and were analyzed using Student’s unpaired t-test, where p < 0.05 is significant.

To determine the impact of increased TNAP expression on the neuroimmune axis *in vivo*, we utilized a transgenic mouse model with VE-cadherin-Cre driven conditional overexpression of TNAP in endothelial cells, or VE-cOE mice. The high expression of TNAP in brain endothelial cells compared to other endothelial cell populations allowed us to focus on the neuroimmune axis. In sham-injured animals, TNAP enzyme activity exhibited a trending increase toward significance in brain tissue (Fig. 10a) and was significantly increased in plasma (Fig. 10b) in VE-cOE mice compared to controls. Next, VE-cOE mice and their controls were subjected to CLP and euthanized 48 hours later. VE-cOE mice had 100% survival (Fig. 10c) and lower clinical scores (Fig. 10d) compared to wild-type controls. Assessment of locomotor activity one day after CLP showed that both VE-cOE and wild-type mice exhibited similar levels of horizonal movement during the first 20 minutes of testing, while significant differences in movement emerged during the final 10 minutes of testing (Fig. 10e). Activity during this period was significantly greater in VE-cOE mice compared to negligible movement in controls (Fig. 10f).

## Discussion

In this study, we investigated a novel immunoregulatory role for TNAP in a mouse model of experimental sepsis. Our results highlight a novel and important role for sustained TNAP enzyme activity at the brain-immune axis. First, we confirmed that SBI-425 does not cross an intact BBB in healthy mice. Second, we found that SBI-425 administration does not affect survival or behavioral outcomes after experimental sepsis. Third, we identified a critical role for TNAP in splenic T cells as we found that SBI-425 downregulates Foxp3+ CD25− populations of CD4+ and CD8+ T cells. Fourth, our results demonstrate that SBI-425 can traverse a compromised BBB as shown by reduced *in vivo* brain TNAP activity following experimental sepsis. Fifth, we showed that SBI-425 potentiates the inflammation-induced loss of barrier function in brain endothelial cells – the cells which interface with the systemic circulation at the BBB. Finally, we demonstrated that overexpression of TNAP in endothelial cells improves survival, clinical scores, and behavioral outcomes associated with early sepsis.

**Figure 11:**
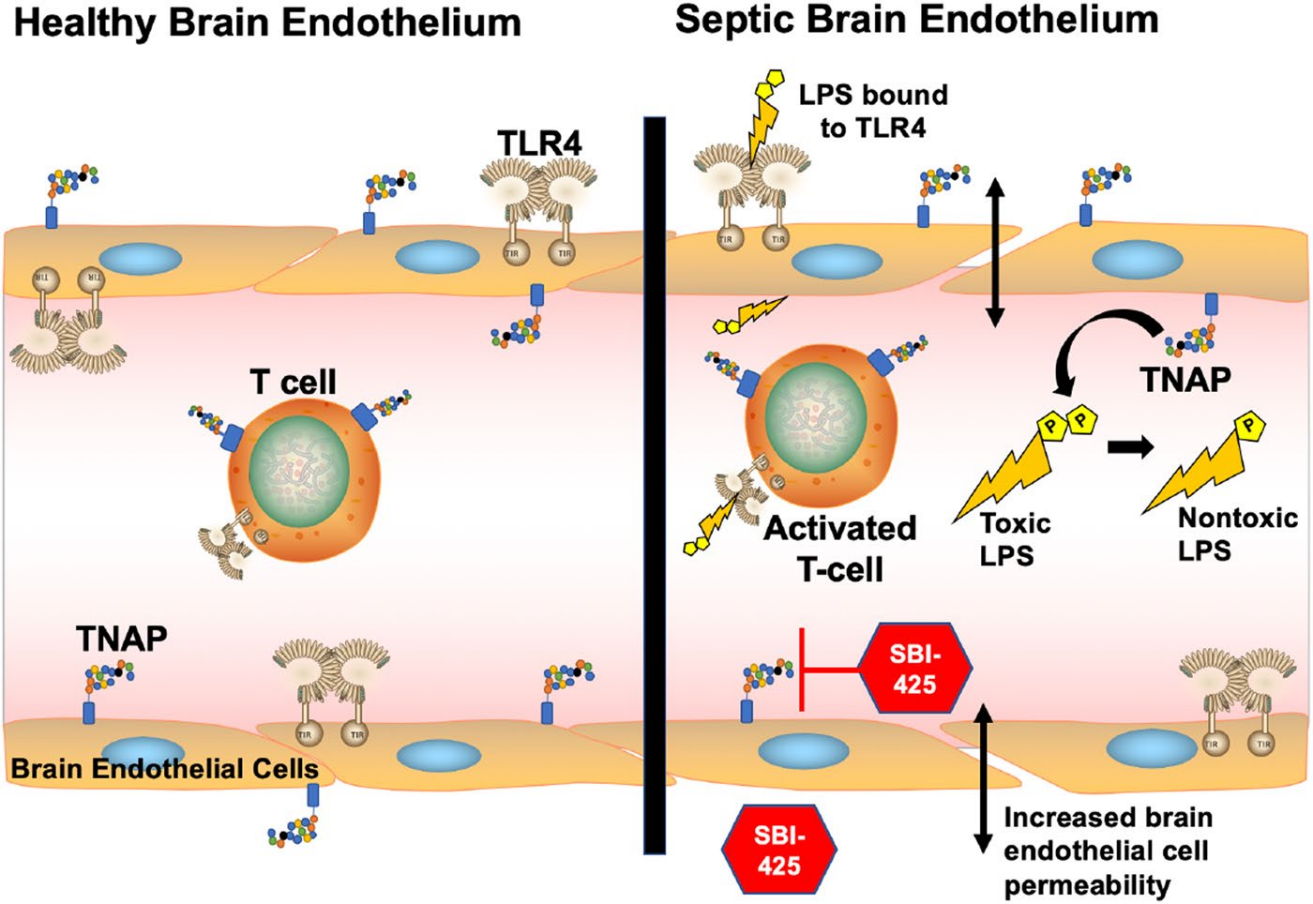
Proposed mechanism of action of TNAP enzyme activity and its inhibition in sepsis. In healthy brain endothelium (left) TNAP performs a role in immune surveillance and the maintenance of BBB permeability. The brain endothelial cells that form the BBB maintain a tight barrier via intact junctional and cytoskeletal proteins which keep toxins and extracellular molecules from entering the brain parenchyma. In sepsis (right), release of systemic cytokines and activation of pro-inflammatory pathways increases brain endothelial cell permeability. In systemic bacterial infections, lipopolysaccharide (LPS) binds to toll-like receptors (primarily TLR4) and initiates a pro-inflammatory innate immune response. One of TNAP’s physiological roles is to dephosphorylate LPS, which converts the molecule from a toxic form to nontoxic form to help reduce inflammation. When TNAP’s enzyme activity is inhibited using SBI-425, more LPS is available to bind to its receptors as well as diffuse across the brain endothelium to activate TLRs on brain resident cells such as microglia and astrocytes.

The translational implications of our results are summarized in Fig. 11. Under normal conditions, TNAP serves a critical role in immune surveillance by maintaining the integrity of the BBB. Under sepsis and other systemic inflammatory conditions, TNAP protects against the loss of BBB permeability as demonstrated using both *in vivo* and *in vitro* inhibition of TNAP via SBI-425 treatment in the current study. Additionally, many other studies have also shown that TNAP dephosphorylates LPS into a nontoxic form^8,28^, which inhibits LPS binding to the toll-like 4 receptor (TLR4) and prevents signaling of TLR4-mediated pro-inflammatory signaling pathways^29,30^. Therefore, TNAP serves a role that is two-fold with regard to endotoxins by: 1) dephosphorylating LPS and 2) preventing the movement of LPS across the endothelium and into the brain parenchyma. Collectively, our results underscore a beneficial role for TNAP within the neuroimmune axis.

The finding that SBI-425 does not cross the BBB in healthy mice is largely supported by the LC/MS/MS result of low SBI-425 brain:plasma ratios following SBI-425 IP administration. Although we did observe decreased AP activity following SBI-425 in some experiments, this decrease may be due to inhibition of AP on the apical side of the BBB rather than in the neural parenchyma. Because SBI-425 binds in a reversible manner, homogenization of brain tissue could release bound inhibitor which could subsequently interact with AP from the parenchyma. Since our methods require homogenization of brain tissue, we were not able to distinguish the cerebrovascular origin of AP from other brain cell types that also express functional TNAP enzymes, which may include neurons, astrocytes, microglia, pericytes, and brain-resident macrophages such as perivascular macrophages. Taken together, this also suggests that SBI-425 may be a valuable pharmacological tool to study TNAP function located on the apical, or luminal, side of the BBB.

At seven days post-sepsis, we measured a significant increase in AP activity in brain homogenate of SBI-425-treated mice and no significant changes between vehicle or SBI-425 treatment in plasma and bone. There are several explanations for this observation. The final SBI-425 IP injection occurred ~18–20 h prior to termination. The half-life of an orally administered 10 mg/kg dose of SBI-425 is 2.3 hr^4^, so the IP-administered SBI-425 in our study should have been metabolized in plasma and bone by 18 hr. In contrast, the increase in brain AP activity may have been caused by a potential positive-feedback mechanism causing an increase in AP protein and/or an increase in activity within specific brain regions when the enzyme is inhibited. Since the entire brain was homogenized, we were unable to address brain region specific alterations in AP activity. However, histological experiments showed a decrease in TNAP activity in brain parenchyma from cortical tissue sections. We focused on the cortex because brain AP activity levels have been characterized in this region^31–34^. Specific physiological functions and mechanistic pathways that regulate TNAP activity remain poorly understood in many organs, including the brain. Brain-localized TNAP has been suggested to aid in proliferation and migration of the developing nervous system^35,36^, promote axonal growth^37^, and regulate the formation and maturation of synapse^31,38^. Additional studies suggest that changes in brain TNAP expression may contribute to Alzheimer’s disease (AD)^39^ and epilepsy^40,41^. TNAP has varied levels of expression in the brain^16,31^, and the amount of total TNAP protein and catalytically active enzyme activity are not equal^42^. Therefore, it is important to quantify the amount of active enzyme and not just the amount of TNAP protein in the brain.

Sepsis is characterized by a hyper-inflammatory, elevated immune response during early sepsis followed by the transition to a hypo-inflammatory, immunosuppressive period in late sepsis prior to restoration of homeostasis^43^. While the innate immune response predominates during early sepsis, the innate and adaptive immune responses function in parallel during later sepsis to reduce immunosuppression and restore immune homeostasis. The immune imbalance can lead to lessened cytokine production and reduced T cell proliferation^44,45^. TNAP is expressed on immune cells, including neutrophils and T lymphocytes, where it plays a role in B cell differentiation, so it is often upregulated at the site of inflammation^46–49^. TNAP has also been shown to modulate T cell function in a heterozygous TNAP+/− mouse model of colitis^50^. We assessed differences in T-cell splenocyte populations at seven days post-sepsis from mice who received either SBI-425 or vehicle treatment. Our flow cytometry data revealed that inhibition of TNAP resulted in differential expression of splenocyte T-cell populations following sepsis. We found decreased mean fluorescence intensity of both CD4+ and CD8+ T-cell subsets in SBI-425-treated compared to vehicle-treated animals. Sepsis is associated with T-cell dysfunction, and our results suggest that TNAP inhibition may enhance T-cell mediated immunosuppression in late sepsis^51,52^. The increase in CD4 + Foxp3+ and CD8 + Foxp3+ Tregs in vehicle- compared to SBI-425- treated mice is consistent with previous findings which show that elevated Tregs are associated with increased survival and improved sepsis outcomes^53^.

Histological analyses of septic mice treated with vehicle or the TNAP enzyme inhibitor, SBI-425, revealed decreased enzyme activity in the neural parenchyma of treated mice. Neuronal processes coursing through the parenchyma appeared more affected by SBI-425 than enzyme activity localized to blood vessels. Regions of blood vessels were intensely stained in both treatment groups; however, distal regions of the same vessel could be either lightly stained, or not apparent in the enzyme stain. The reasons for these differences are not well understood but may reflect differences in regional exposure to blood products produced by the sepsis surgery, or whether levels of the inhibitor were sufficient to downregulate TNAP enzyme activity. Double-staining for TNAP enzyme activity and GFAP immunoreactivity revealed regions of vessels that were co-stained, adjacent to regions that were singly stained for TNAP activity or GFAP. The mechanisms regulating differential staining are not known. However, the loss of barrier function in murine primary brain endothelial cells treated with SBI-425 demonstrates a significant role for the preservation of TNAP enzyme activity in cerebral microvessels. The BBB-like properties of brain endothelial cells were diminished in the presence of SBI-425 on day 1 compared to IFNγ + TNFα treated counterparts. SBI-425 treatment also potentiated the long-term effects of an IFNγ + TNFα inflammatory stimulus, suggesting that inhibition of TNAP enzyme activity during acute pro-inflammatory conditions may have highly detrimental effect. These results are supported by previous work which demonstrates a loss of brain endothelial cell permeability in cells treated with levamisole, a pan-AP inhibitor^42,54^.

Glial reactivity of astrocytes and microglia reflected a residual sepsis effect. Although there were no significant differences between vehicle and SBI-425 treatment, these results have important implications for elucidating a neuroinflammatory role for TNAP in both early and late sepsis. The seven-day time course of experimental sepsis and tissue harvest occurred after the typical early peak response of microglia, but prior to the relatively late peak response of astrocytes. During the stage of late sepsis examined in this study, most microglia displayed surveillance morphology. In contrast, activated, phagocytic cells were observed in the vicinity of blood vessels, where interaction with residual blood components from the sepsis surgery could still manifest. Likewise, astrocytes displayed an activated morphology with elevated levels of GFAP, but no effect of the inhibitor was observed. High-magnification microscopy also revealed the presence of small, clear vesicles in the processes of astrocytes. These vesicles are presumed to be exocytic vesicles prior to release that represent chemical communication between astrocytes and neighboring cells. Astrocytic release of extracellular vesicles has recently emerged as a major mechanism of communication in neuroinflammation and neurodegeneration and may contribute to bidirectional communication within the neuroimmune axis^55,56^.

Results from the *in vivo* model of experimental sepsis suggest that SBI-425 may disrupt barrier function under physiological conditions when BBB integrity is compromised. This finding was corroborated by *in vitro* barrier function studies in murine BMECs. Although we did not observe changes in locomotor activity in experimental sepsis, SBI-425 could affect and potentiate behavioral changes in other domains associated with sepsis including: learning and memory, nociception, or depression. In contrast, we observed that overexpression of TNAP in endothelial cells improved neurobehavioral outcomes. These results are important because altered mental status is one of the most common and severe symptoms associated with sepsis and its underlying origins have been linked to BBB dysfunction^57,58^.

In summary, our results show that the highly-specific TNAP inhibitor, SBI-425, is unlikely to cross an intact BBB in the absence of injury or disease. In contrast, *in vivo* and *in vitro* results demonstrate that SBI-425 can traverse the BBB and disrupt TNAP activity in the brain parenchyma during injury or disease. However, the long-term consequences, if any, of a short-term disruption of brain TNAP activity are unclear and will be investigated in future studies. This is important because short-term perturbations in cerebrovascular function have been hypothesized to have long-term implications for neurological function. Thus, the short-term loss of TNAP activity in brain endothelial cells during sepsis may be one reason for diminished neurological function observed in sepsis survivors. As SBI-425 is currently under investigation as a therapeutic agent to treat vascular calcification^6^, caution may be warranted when individuals undergoing SBI-425 therapy experience co-morbid pathological conditions associated with the loss of BBB integrity. Individuals with ischemic stroke, traumatic brain injury, sepsis, and Alzheimer’s disease represent populations where SBI-425 could easily cross the BBB^59–61^. TNAP has many unexplored important physiological roles in the nervous system and the immune system which rely on the tightly controlled regulation of its enzymatic activity in different cells and tissues. Thus, the availability of a novel and highly-specific pharmacological compound such as SBI-425 provides a valuable tool to elucidate novel, *in vivo* roles for TNAP within the brain-immune axis.

## Materials and Methods

### Animals

Male and female C57BL/6J mice were obtained from The Jackson Laboratory (Bar Harbor, ME, USA) and bred in the West Virginia University Office of Laboratory Animal Resource (OLAR) facility. B6.FVB-Tg(Cdh5-cre)7Mlia/J mice expressing Cre recombinase under the control of the endothelial cell-specific Cdh5 promoter (also known as VE-Cadherin-Cre or VE-Cre) were obtained from The Jackson Laboratory (Bar Harbor, ME, USA; stock 006137)^62–64^. Hprt^ALPL^ knock-in mice were previously described^65^. VE-Cre male mice were bred with homozygous female Hprt^ALPL^ mice to create mice with elevated expression, i.e. overexpression, of TNAP in endothelial cells (i.e. VE-cOE). Animals were genotyped by PCR using DNA extracted from ear snips using the Purelink Genomic DNA Mini Kit (Invitrogen, Carlsbad, CA, USA), then a Veriti 96-well Thermal Cycler (Applied Biosystems). VE-Cre specificity was determined using forward and reverse primers GAACCTGATGGACATGTTCAGG/AGTGCGTTCGAACGCTAGAGCCTGT; and PCR conditions 94 °C for 1 min, [(94 °C for 30 sec, 60 °C for 30 sec, 72 °C for 45 sec) × 40 cycles], then 72 °C for 1 min (band length 320 kb). TNAP overexpression-specificity was determined using two sets of forward and reverse primers: AATGCCCAGGTCCCTGACAGC/GGTTCCAGATGAAGTGGGAGT (presence of 505 kb band indicating overexpression) and TGTCCTTAGAAAACACATATCCAGGG/CTGGCTTAAAGACAACATCTGGGAGA (presence of 345 kb band indicating wild-type gene); and PCR conditions 94 °C for 1 min, [(94 °C for 30 sec, 68 °C for 30 sec, 72 °C for 45 sec) × 40 cycles], then 72 °C for 1 min. Wild-type mice of the same genetic background strains (VE-Cre and Hprt^ALPL^) were used as control animals. All mice used were aged 2–4 months. All animal studies were approved by the Institutional Animal Care and Use Committee of West Virginia University and were in compliance with the National Institutes of Health guidelines for the humane treatment of animals.

### SBI-425 preparation

SBI-425 was synthesized as previously described^24^. SBI-425 powder was dissolved using heat and sonification in 100% dimethyl sulfoxide (DMSO, Sigma-Aldrich, Milwaukee, WI) to make a 12.5 mg/ml stock solution stored at −80 °C. For *in vivo* administration of SBI-425, the stock was diluted to 1 mg/ml in a vehicle solution comprised of: 10% DMSO, 10% Tween-80 (Sigma-Aldrich), and 80% water. All control mice were injected with a volume of vehicle solution equivalent to the largest volume of SBI-425 administered to all mice in that cohort.

### Sample collection for biochemical studies

Mice were anesthetized with isoflurane and euthanized by cardiac perfusion with 0.9% saline. Plasma was collected by cardiac puncture into Microvette tubes coated with lithium-heparin (Sarstdet; Nümbrecht, Germany), centrifuged at 10,000 × g for 5 mins, aliquoted, and stored at −80 °C until analysis. Brain cortices (excluding olfactory bulbs and cerebellum) and bone (femur and/or tibia) tissues were collected following saline perfusion, homogenized in AP buffer (comprised of: 1 M Tris-HCl (Tris Base: Fisher Scientific, Pittsburg, PA; Hydrochloric acid: Sigma-Aldrich, Milwaukee, WI), 1 M MgCl_2_ (Fisher Scientific), 50 mM ZnCl_2_ (Acros Organics, NJ), and deionized water) without the substrate, and stored at −80 °C for further analysis. Samples were spiked with 40 μM SBI-425 (equivalent to a 10 mg/kg dose), 100 μM of an unoptimized TNAP inhibitor (TNAPI; MLS-0038949, Cat #: 613810, Millipore-Sigma, Burlington, MA^25^), or vehicle (DMSO) and AP activity was detected using the methods described below.

### Dosing and sample collection for *in vivo* studies

Mice were weighed immediately prior to treatment injections to ensure accurate dosing of each animal, and sacrificed 1, 4, and 6 hours post-treatment for 25 mg/kg intraperitoneal (IP) administration, or 10, 30, and 60 minutes post-treatment for 5 mg/kg intravenous (IV) injection via retro-orbital administration. Vehicle-treated animals were sacrificed at the latest time-point (6 hours post IP injection and 1 hour post IV injection). Mice were anesthetized with isoflurane and euthanized by cardiac perfusion with 0.9% saline. Plasma was collected by cardiac puncture into Microvette tubes coated with lithium-heparin, centrifuged at 10,000 × g for 5 mins, aliquoted, and stored at −80 °C until analysis. Organ tissues were collected following saline perfusion, homogenized in AP buffer without the substrate, and stored at −80 °C.

### Detection of alkaline phosphatase (AP) activity

AP activity assays were adapted from a previously published protocol and performed in a 384-well plate^66^. Briefly, plasma and tissue homogenates were centrifuged to remove insoluble material, diluted if necessary, and then mixed with a buffer comprised of 1 M Tris-HCl (Tris Base: Fisher Scientific, Pittsburg, PA; Hydrochloric acid: Sigma-Aldrich, Milwaukee, WI), 1 M MgCl_2_ (Fisher Scientific), 50 mM ZnCl_2_ (Acros Organics, NJ), deionized water, and 13.5 mM *para*-nitrophenylphosphate (pNPP; Millipore Sigma, Billerica, MA) solutions. Brain homogenates were diluted 1:10 using AP buffer with pNPP omitted. Each sample was plated in duplicate and matched to a background control using a control buffer containing 5% of a 200 mM sodium orthovanadate (BeanTown Chemical, Hudson, NH) solution. AP activity levels were read kinetically every 5–10 mins at O.D. 380 nm for 5 hours on a Synergy H1 Hybrid Reader and absorbance was acquired with Gen5 Version 2.01.14 software (BioTek Instruments, Inc., Winooski, VT). The area under the curve (AUC) was calculated to determine TNAP activity levels in each sample.

### Liquid chromatography tandem mass spectrometry (LC/MS/MS)

Mass spectrometry analysis was performed on SBI-425 (10 mg/kg IP dose; n = 3 per group) in plasma and homogenized brain of male C57BL/6 mice harvested at 2 hr and 8 hr after injection. For plasma preparation, an aliquot of 60 µL sample was protein precipitated with 480 µL internal standard solution, vortexed for 1 min, and centrifuged at 13000 rpm for 15 min. Brain homogenate was prepared by homogenizing brain with 4 volumes (w:v) of homogenizing solution (deionized water). An aliquot of 60 µL sample was protein precipitated with 480 µL internal standard solution, the mixture was vortex-mixed for 1 min and centrifuged at 13000 rpm for 15 min. 5 µL supernatant was injected for analysis using the Linear Ion Trap Quadrupole LC/MS/MS Mass Spectrometer (AB Sciex Instruments, Model #1004229, API 4000). Internal standards of 100 ng/mL dexamethasone, 100 ng/mL diclofenac, and 100 ng/mL tolbutamide in acetonitrile were used.

### Cecal ligation and puncture

A sublethal model of experimental sepsis was induced by cecal ligation and puncture (CLP) as previously described (Vachharajani *et al*., 2014). Briefly, on day 0 C57BL/6J female mice (n = 25) were anesthetized with isoflurane in normal air (3% induction, 1% maintenance), numbed with a topical 2% lidocaine hydrochloride solution (Pheonix, St. Joseph, MO) applied to the peritoneum, and subjected to CLP surgery. CLP surgery was completed by tying off two-thirds of the cecum using a silk thread suture (Look Surgical Specialties, Reading, PA), poking two holes into the cecum with a 22-gauge needle and squeezing a small amount of fecal matter up to the tissue surface. The cecum was returned to the abdominal cavity, and the incision was closed using 6-0 (peritoneum) and 5-0 (skin) monofilament (Unify PGA Surgical Sutures, Sunnyvale, CA). After completion of the surgery, mice were given 1 ml subcutaneous normal saline for rehydration. One hour following surgery, and once daily for 7 days total, mice were IP injected with 25 mg/kg SBI-425 (n = 10) or vehicle (n = 12); this SBI-245 dose is similar to previously reported values in other published studies^4,67^. Weights, temperatures, and clinical assessments were performed daily between 9:00 am and 12:00 pm. Clinical assessments were performed by a blinded investigator and calculated based on a modified murine sepsis scoring system with a range of 0 (no pathology) – 36 (probable death); mice with a score ≥24 were euthanized^68,69^. On day 7 post-surgery (24 hr following the last SBI-425 or vehicle treatment), mice were harvested for plasma and tissues.

### Open field testing

On day 2 post-CLP, mice underwent open field testing to assess locomotor activity using individual 16 × 16 × 15 chambers (Photobeam Activity System, San Diego Instruments, San Diego, CA) measured as the number of beam breaks. Parameters included 5-minute interval assessments of a 60-minute total trial using 3 × 3 periphery.

### Tissue harvest and processing

Mice were deeply anesthetized with isoflurane and were perfused transcardially with a perfusion pump (Masterflex 7524-10, Cole-Parmer, Vernon Hills, IL) set to 5.0 ml/min. Blood was removed with 10 ml 0.9% saline and tissues were fixed with 50 ml of 4% cold paraformaldehyde (Fisher Scientific, Pittsburgh, PA). Brains were removed from the skull, bisected, and post-fixed in 4% paraformaldehyde overnight at 4 °C. The following day, the hemispheres were rinsed in 0.01 M phosphate buffered saline (PBS) and incubated sequentially in 10%, 20%, and 30% sucrose in PBS for 24 hr each.

### Splenocyte isolation

Perfused spleens were collected in 10 ml cold isolation buffer containing RPMI-1640 (Corning, Manassas, VA) supplemented with 1.5% fetal bovine serum (FBS, Gemini Bio-Products, West Sacramento, CA) and processed immediately to obtain a single cell suspension. Briefly, spleens were meshed gently using the back of a plunger and filtered through a 70 µm cell strainer (Fisher Scientific). Cells were collected by centrifugation at 300 × g for 10 min at 4 °C. Red blood cells were lysed with 4 ml of ACK Lysis Buffer (Lonza, Portsmouth, NH) per spleen, incubated for 2–3 min at room temperature (RT) and washed as previously described^70^. Viability and total splenocyte yield were determined by trypan blue (Life Technologies, Eugene, OR) exclusion. Splenocytes were re-suspended to a final concentration of 2.5–3 × 10^6^ cells/ml with FACS buffer (1X PBS, 5 mM EDTA, 2% FBS) for flow cytometry.

### Flow cytometry

Immunophenotyping of splenocytes was performed by flow cytometry. Briefly, cells were washed twice in sodium azide- and protein-free cold PBS and stained with fixable viability dye eFluor780 (Cat. #65-0865; Thermo Fisher Scientific, Waltham, MA) for 30 min at 4 °C in the dark. Cells were then briefly washed with FACS buffer and blocked with Ultra-Leaf purified anti-mouse CD16/32 (Clone: 93; Cat. #101330; BioLegend, San Diego, CA) for at least 20 mins. Following non-specific blocking, cells were stained with monoclonal antibodies for CD45-PE (Clone: REA737; Cat #: 130-110-659), CD11b-VioBlue (Cat #: 130-097-336), CD11c-PerCp-Vio700 (Cat #: 130-103-806), Ly6G-APC (Order #130-107-914), Ly6C-PE-Vio770 (Cat #: 130-103-046) from Miltenyi Biotec (Auburn, CA USA) or anti CD4-FITC (Clone: RM4-5; Cat #: 11-0042-82), CD25-PE (Clone: PC61.5; Cat #: 12-0251-82), CD19-PerCp-Cy5.5 (Clone: 1D3; Cat. #:45-0193-80) and CD8a-eFluor450 (Clone: 53-6.7; Cat#: 48-0081-82) from Thermo Fisher Scientific for 10 min at 4 °C. Appropriate single stained controls were prepared for fluorophore compensation using compensation beads (Invitrogen, Carlsbad, CA). For intracellular FOXP3 detection, after staining the surface markers (CD4 or CD25), cells were fixed/permeabilized using Mouse regulatory T cell staining kit according to manufactory’s instruction (Invitrogen) and stained with anti-FOXP3-PE-Cy5 (Clone: FJK-16s; Cat #:15-5773-82). Fluorescent assessment was carried out with BD LSR Fortessa using FACS Diva software (BD Biosciences, San Jose, CA). Single cells were identified by forward scatter and side scatter, and viable cells were gated. Cells were gated for CD45 positive populations then divided into lymphoid cells, which included B cells (CD11c-CD19+), T-helper cells (CD3 + CD4+), cytotoxic T cells (CD3 + CD8+), T-regulatory cells (CD4 + CD25 + Foxp3+); and myeloid cells, which included neutrophils (Ly6G+), monocytes (Ly6C+), and dendritic cells (CD11c+). All data were compensated and spectral overlap was minimized using the automatic compensation feature in BD FACSDiva software (BD Biosciences). The gating strategy for lymphocytes is shown in Supplementary Fig. 1.

### Embedding and sectioning

Hemispheres were co-embedded into a 15% gelatin matrix in groups of nine for simultaneous sectioning. The gelatin block was processed sequentially through 4% paraformaldehyde for 24 hr, 15% sucrose for 24 hr, and 30% sucrose for 48 hr. The block was trimmed and quick frozen in dry ice/isopentane for three min. Sectioning was performed in the coronal plane at 40 µm on a sliding microtome (HM 450, ThermoFisher Scientific) equipped with a 3 × 3 freezing stage (BFS-40MPA, Physitemp, Clifton, NJ) at −20 °C. Sections were collected into a series of six cups filled with PBS + sodium azide (0.6 g sodium azide/1 L PBS). Adjacent cups were used for sequential stains or immunostains.

### Antibodies and immunohistochemistry

The following antibodies were used (antibody names, registry numbers, and dilutions): glial fibrillary acidic protein (GFAP) - 10013382 (Z0334, Dako/Agilent; 1:10,000 primary, 1:10,000 secondary); Iba-1-839509 (019–19741, Wako; 1:2,000 primary, 1:1,000 secondary). Sections were stained free-floating using a modified ABC procedure (Vector Laboratories, Burlingame, CA). Sections were treated with 10% methanol, 10% hydrogen peroxide in Dulbecco’s modified phosphate buffered saline (DPBS; 136 mM NaCl, 8 mM Na_2_HPO_4_, 2.6 mM KCl, 1.5 mM KH_2_PO_4_) for 15 min to quench endogenous peroxidase. Following three rinses in DPBS for five min each, sections were incubated in a permeabilizing solution (1.8% L-lysine, 4% normal horse serum, 0.2% Triton X-100 in DPBS) for 30 min at room temperature. Sections were transferred directly to primary antibody solution in DPBS + 4% normal horse serum and were incubated overnight at room temperature. The following day, sections were rinsed three times in DPBS for five min each and transferred to secondary antibody solution in DPBS + 4% normal horse serum for two hours at room temperature. Following three rinses in DPBS for five min each, sections were incubated in Avidin D-HRP (1:1000 in DPBS, Vector Laboratories, Burlingame, CA) for 1 h at room temperature, rinsed three times in DPBS for five min each, and incubated with chromogen solution (3-3′ diaminobenzidine, 50 mg in 100 ml DPBS + 50 µl 30% hydrogen peroxide, Electron Microscopy Sciences, Hatfield, PA) for five min. Sections were rinsed three times in DPBS for five min each, mounted onto microscope slides (Unifrost+, Azer Scientific, Morgantown, PA), air-dried overnight, dehydrated through a standard dehydration series, and cover slipped with Permount (Fisher Scientific). A subseries of sections was double-stained for GFAP by immunohistochemistry and subsequently for TNAP enzyme activity.

### Microscopy and image analysis

Slides were viewed on a Leica DM6B microscope and images were captured using Leica LASX software. Images of GFAP immunoreactivity were captured at 40X extended depth of field (EDOF) in CA1 of hippocampus. Images of ALPL enzyme staining and Iba-1 immunoreactivity were captured at 20X EDOF in medial orbital cortex. For GFAP, images were captured from CA1 in hippocampus of three adjacent sections at 40X. A batch processing file was programmed in Photoshop with the following steps: (1) convert to grayscale; (2) invert; (3) threshold = 144; (4) select All; (5) record measurement – mean gray scale. Mean intensity values were collected and analyzed by unpaired t-test. For Iba-1, images were captured from medial orbital cortex of three adjacent sections at 20X. A batch processing file was programmed in Photoshop with the following steps: (1) convert to grayscale; (2) invert; (3) threshold = 164. Cell bodies were counted manually using the count tool and were recorded and analyzed in Prism 7.0.

### Alkaline phosphatase enzyme activity histology and image analysis

Brains were evaluated for alkaline phosphatase enzyme activity by staining free-floating with a BCIP/NBT substrate kit as described (SK-5400, Vector Laboratories, Burlingame, CA). Sections were rinsed in DPBS twice for 5 min each, once in 0.1 M Tris-HCl (pH = 9.5) for 5 min, and incubated in the staining solution for 24 hr. Following three rinses in Tris-HCl, the sections were mounted onto microscope slides (Unifrost+, Azer Scientific, Morgantown, PA), air-dried overnight, dehydrated through a standard dehydration series, and cover slipped with Permount. Images were viewed and analyzed in Photoshop CC19.0 (Adobe Systems Inc., San Jose, CA). The density of ALPL enzyme staining was measured from the blue channel. A 2.0 × 2.0 cm square was drawn and moved to ten random positions in between the vasculature of three adjacent sections. The mean intensity of the blue channel was captured from the histogram, and recorded in Excel (Microsoft Corp., Redmond, WA). Density values were subtracted from 255 (the maximum bright level) to reconcile the inverse relationship between reduced staining and increased brightness. The average staining density was calculated for each animal and differences in mean staining intensity were evaluated between treatment groups.

### Brain endothelial cell primary culture

Brain microvascular endothelial cells (BMECs) were cultured based on published protocols with minor modifications^71^. Briefly, five adult male mice (3–4 months) were perfused with ice-cold phosphate-buffered saline. Cortices were dissected and homogenized, followed by tissue digestion in papain and DNase I (both from Worthington Biochemical Corp, Lakewood, NJ) at 37 °C for 1 hr. Following trituration, the homogenate was centrifuged (1360 × g) for 10 minutes, followed by myelin removal. The cell pellet was resuspended in endothelial cell growth medium (ECGM: F12 medium with 10% fetal bovine serum (FBS), endothelial growth supplement, ascorbate (2.5 µg/ml), L-glutamine (4 mM), and heparin (10 µg/ml)), and plated into four collagen-coated wells (calf skin collagen, Sigma-Aldrich) of a six-well plate. Cultures were treated with fresh ECGM medium the next day followed by treatment with puromycin hydrochloride (4 μg/ml) with EGCM + FBS for 2.5 days. Cultures reached confluency after 5–7 days, after which they were detached with Accutase (Innovative Cell Technologies, San Diego, CA) and seeded onto 3 separate collagen-coated 16-well E-Plate 16 PET arrays (Acea Biosciences, San Diego CA) at a concentration of 30,000 cells/well and loaded, in parallel, onto an xCelligence RTCA DP system (ACEA Biosciences) enclosed in a tissue culture incubator. A subset of primary BMECs from each preparation was assessed for purity and found to be >99% as assessed CD31 immunolabeling (data not shown; CD31 goat polyclonal 1:250, Santa Cruz Biotechnology, Santa Cruz, CA).

### Brain endothelial cell barrier function assays

The barrier function of primary BMECs was quantified by continuous recording of impedance in each well, which was recorded at 15-minute intervals and reported as cell index (CI). Cells reached confluence, indicated by a plateau in CI, which was ~25 hours after seeding. At this time, all arrays were removed from the xCelligence RTCA DP system (ACEA Bioscience, San Diego, CA) and duplicate wells in each array were treated with 200 μl of the following: vehicle (DMSO, 0.3%), SBI-425 (100 μM), tumor necrosis factor-α (TNFα, 10 ng/ml) and interferon-γ (IFNγ, 10 ng/ml), or SBI-425 in combination with TNFα + IFNγ; the concentration of DMSO vehicle was equivalent to the highest final concentration of DMSO in all other treatments of SBI-425. The concentration of SBI-425 was selected based on dose-response curves (1 μM to 500 μM) to test the effects of SBI-425 and TNAPI on barrier function. CI was recorded continuously for ~96 hours at 15 min intervals and analyzed with RTCA Software 2.0 (ACEA Bioscience). At the completion of the data collection period, the CI for all wells was normalized to a single timepoint, i.e. ~1 h after treatment (~26 hr total), and calculated over the 96 hr duration of treatment. To calculate the slope of the normalized CI over a 96-hr period, results were divided into 4-separate 24 hr, i.e. 1 day, intervals, and the slope was calculated by RTCA Software using the equation: CI = slope * time + intercept.

### Primary BMEC treatment conditions and reverse-transcriptase PCR

BMECs were plated in collagen-coated 24-well dishes and allowed to reach confluence. Following stimulation for 24 hours with the treatments described above, total RNA was isolated using Qiagen RNAeasy Mini Kit (Qiagen, Germantown, MD) using the manufacturer’s instructions. Total RNA was quantified using a NanoDrop 2000 spectrophotometer (Thermo Scientific, Waltham, MA). 10 ng of total RNA was used to quantify *Alpl* expression using a one-step quantitative real time PCR reaction consisting of Taqman Universal Master Mix II, no uracil-N-glycosylase (UNG), MuLV Reverse Transcriptase, RNase inhibitor, and Taqman Expression Assays for: mouse *Alpl* (Assay ID: Mm03024075_m1) and 18 S (Assay ID: Hs99999901_s1); 18 S was used as a housekeeping gene. Quantitative real time PCR was performed in a 96-well reaction plate (Applied Biosystems) by using a StepOne Plus real time PCR instrument (Applied Biosystems) under the following conditions: reverse transcription: 45 °C for 30 minutes followed by 95 °C for 5 minutes; reverse transcription was followed immediately by 40 cycles of PCR amplification (95 °C for 10 seconds and 60 °C for 1 min). Fold changes in *Alpl* gene expression were calculated using the 2^−ΔΔ Ct^ method and results for treatment groups were expressed as the fold change compared to vehicle-treated (control) cells as previously described^72^.

### Statistical analysis

All results were reported as means ± SEM unless otherwise noted and were analyzed with Student’s unpaired t-test for two comparisons or one-way ANOVA for three or more comparisons, followed by Dunnett’s test for comparisons made to a control group or Tukey’s test for comparisons made between all groups. Survival curve analysis was performed by using log rank (Mantel-Cox) test. Longitudinal analyses for weight change and clinical score were analyzed by using a mixed effects model (restricted maximum likelihood, REML) followed by Dunnett’s multiple comparisons test. Each experiment was performed with a minimum of n = 3 mice for *in vivo* studies or n = 3 technical replicates for *in vitro* studies. Specific details for each experiment are provided in the figure legends. All data were analyzed using GraphPad Prism 8.0 software (GraphPad Inc., La Jolla, CA) with alpha set to 0.05 as the significance threshold. Significance determined from post hoc testing was designated by *P ≤ 0.05; **P ≤ 0.01; ***P ≤ 0.001; and ****P ≤ 0.0001.

## Supplementary information

Supplementary Information (SI)
