# Supplementary material for: Systemic inhibition of tissue-nonspecific alkaline phosphatase alters the brain-immune axis in experimental sepsis
Source: Sci Rep. 2019 Dec 11;9:18788. doi: 10.1038/s41598-019-55154-2 (PMC6906465; doi:10.1038/s41598-019-55154-2)
Supplement: Supplementary file 1 — Supplementary Information (SI) [file 41598_2019_55154_MOESM1_ESM.docx]

**Systemic inhibition of tissue-nonspecific alkaline phosphatase alters the brain-immune axis in experimental sepsis**

Allison L. Brichacek, Stanley A. Benkovic, Sreeparna Chakraborty, Divine C. Nwafor, Wei Wang, Sujung Jun, Duaa Dakhlallah, Werner J. Geldenhuys, Anthony B. Pinkerton, José Luis Millán, Candice M. Brown

**
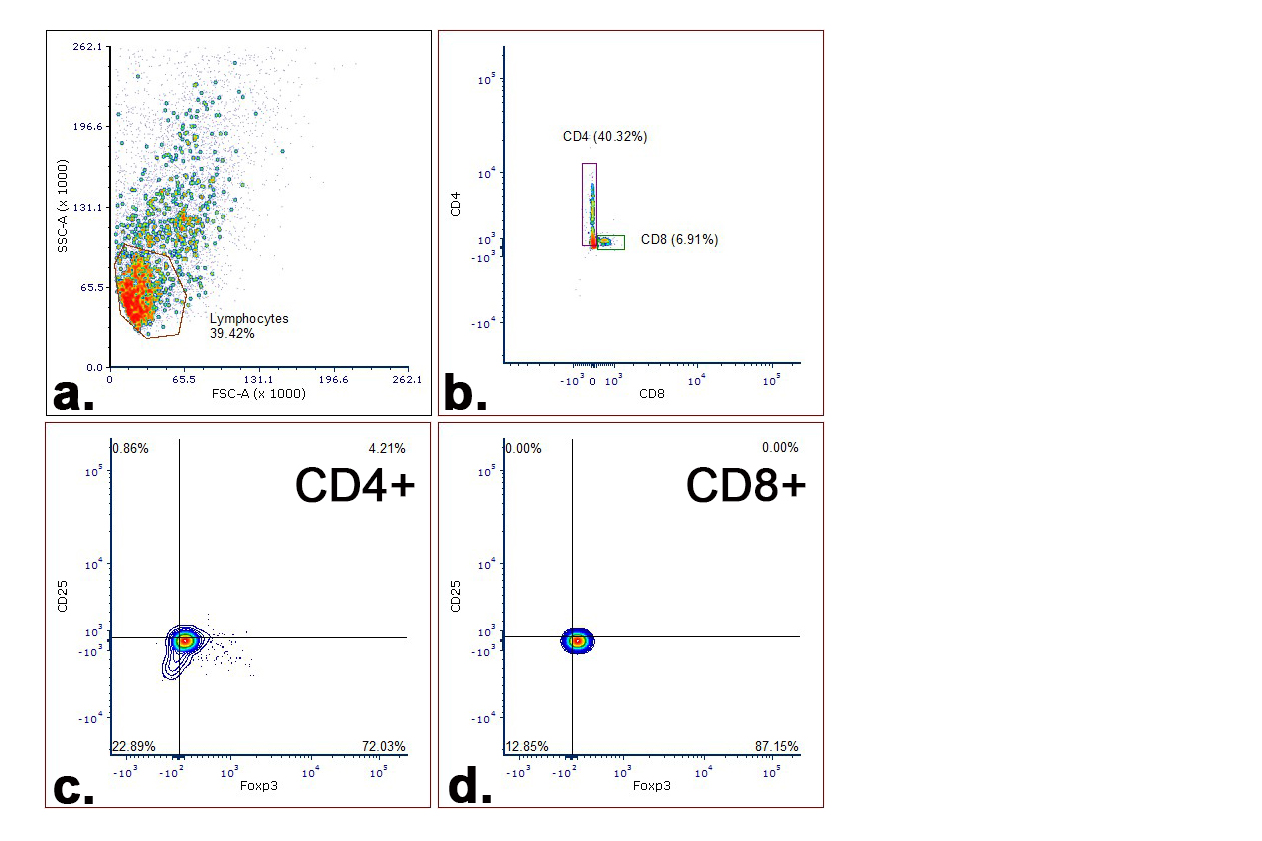
**

**Supplemental Figure 1. Gating strategy for murine splenocytes.** (**a**) Murine splenocyte CD45+ cells were first gated as CD3+ cells. (**b**) CD3+ cells were then gated for T-helper cells (CD3+CD4+) and cytotoxic T cells (CD3+CD8+). **(c)** CD4+ cells were further gated as: regulatory T cells (Tregs: CD3+CD4+Foxp3+CD25+), Foxp3+ T cells (CD3+CD4+Foxp3+CD25-), CD25+ T cells (CD3+CD4+CD25+Foxp3-/low), and non-Tregs or naïve/resting T cells that did not express any CD25+Foxp3+ markers (CD3+CD4+CD25-Foxp3-). (**d**) The same strategy was applied to CD8+ T cells to gate for CD8+ Tregs.


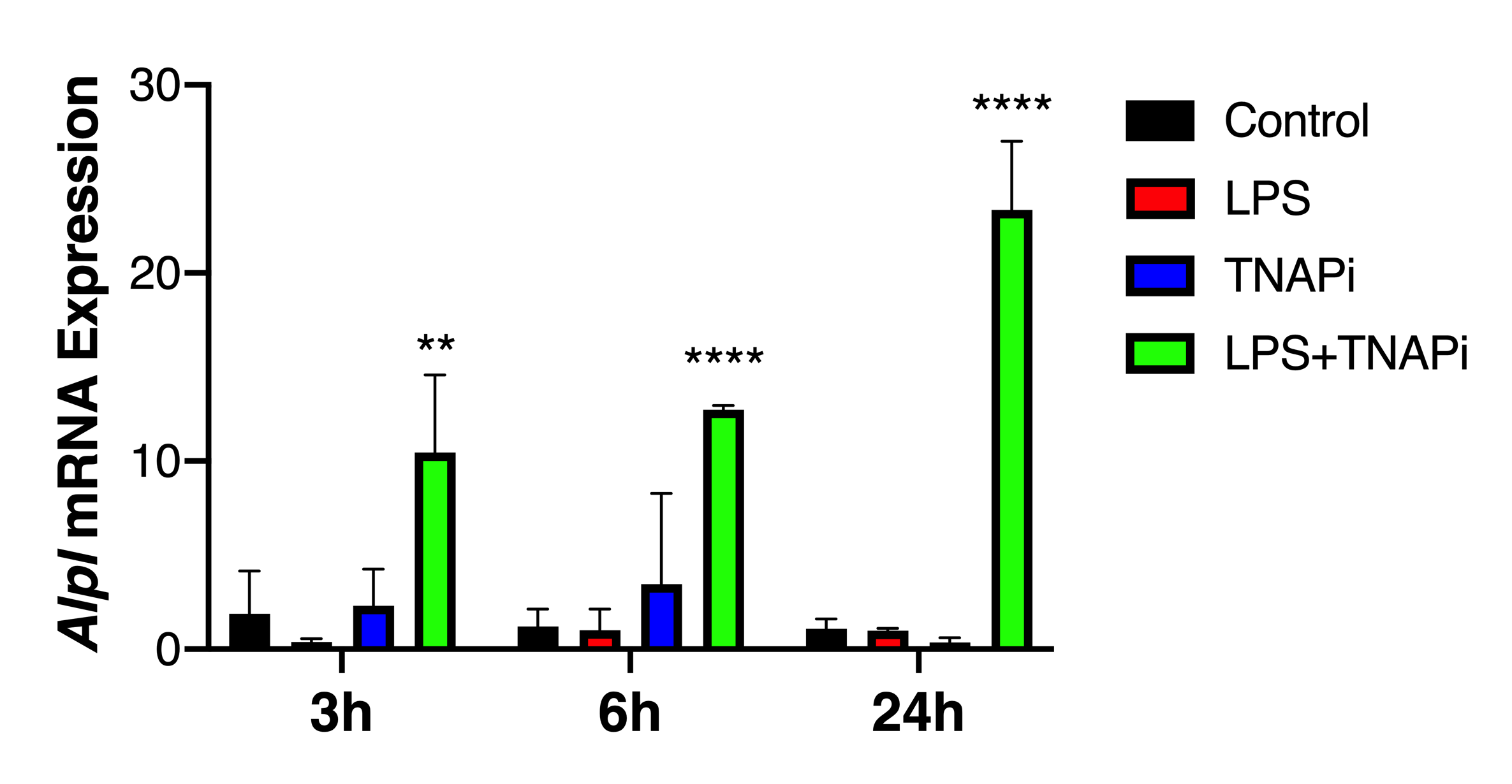


**Supplemental Figure 2. Relative expression of TNAP in murine BMECs.** C57BL/6 mouse (6-8 weeks old) primary BMECs showed increased TNAP (*Alpl*) mRNA expression by 3 to 24 hrs post-stimulation with a combination treatment of LPS and TNAPi. Data were analyzed using two-way ANOVA followed by Tukey’s multiple comparisons test. Significance is compared to the control group, where **p=0.002 and ****p<0.0001.
